# The Performance and Clinical Applicability of HER2 Digital Image Analysis in Breast Cancer: A Systematic Review

**DOI:** 10.3390/cancers16152761

**Published:** 2024-08-03

**Authors:** Gauhar Dunenova, Zhanna Kalmataeva, Dilyara Kaidarova, Nurlan Dauletbaev, Yuliya Semenova, Madina Mansurova, Andrej Grjibovski, Fatima Kassymbekova, Aidos Sarsembayev, Daniil Semenov, Natalya Glushkova

**Affiliations:** 1 Department of Epidemiology, Biostatistics and Evidence-Based Medicine, Al-Farabi Kazakh National University, Almaty 050040, Kazakhstan; 2 Rector Office, Asfendiyarov Kazakh National Medical University, Almaty 050000, Kazakhstan; kalmatayeva.z@kaznmu.kz; 3 Kazakh Research Institute of Oncology and Radiology, Almaty 050022, Kazakhstan; dilyara.kaidarova@gmail.com; 4 Department of Internal, Respiratory and Critical Care Medicine, Philipps University of Marburg, 35037 Marburg, Germany; nurlan.dauletbaev@mcgill.ca; 5 Department of Pediatrics, Faculty of Medicine and Health Sciences, McGill University, Montreal, QC H4A 3J1, Canada; 6 Faculty of Medicine and Health Care, Al-Farabi Kazakh National University, Almaty 050040, Kazakhstan; 7 School of Medicine, Nazarbayev University, Astana 010000, Kazakhstan; yuliya.semenova@nu.edu.kz; 8 Department of Artificial Intelligence and Big Data, Al-Farabi Kazakh National University, Almaty 050040, Kazakhstan; madina.mansurova@kaznu.edu.kz; 9 Central Scientific Research Laboratory, Northern State Medical University, Arkhangelsk 163000, Russia; a.grjibovski@yandex.ru; 10 Department of Epidemiology and Modern Vaccination Technologies, I.M. Sechenov First Moscow State Medical University, Moscow 105064, Russia; 11 Department of Biology, Ecology and Biotechnology, Northern (Arctic) Federal University, Arkhangelsk 163000, Russia; 12 Department of Health Policy and Management, Al-Farabi Kazakh National University, Almaty 050040, Kazakhstan; 13 Department of Public Health and Social Sciences, Kazakhstan Medical University “KSPH”, Almaty 050060, Kazakhstan; f.kassymbekova@gmail.com; 14 School of Digital Technologies, Almaty Management University, Almaty 050060, Kazakhstan; a.sarsembayev@almau.edu.kz; 15 Health Research Institute, Al-Farabi Kazakh National University, Almaty 050040, Kazakhstan; glushkovanatalyae@gmail.com; 16 Computer Science and Engineering Program, Astana IT University, Astana 020000, Kazakhstan; daniil.semenov@nu.edu.kz

**Keywords:** breast cancer, HER2, digital image analysis, performance evaluation, applicability in real clinical practice

## Abstract

**Simple Summary:**

HER2-positive breast cancer occurs in 15–30% of cases and has a poor prognosis. Digital image analysis of HER2 is promising, but its implementation in real clinical practice remains unclear. This systematic review evaluates the effectiveness of digital image analysis algorithms for HER2 in breast cancer and their performance, with a focus on testing them in real-world clinical settings. The authors aim to assess the applicability of these algorithms in practical clinical scenarios. By analyzing 25 papers from the period 2013–2024 and emphasizing mostly deep learning approaches, the review underscores the importance of standardized evaluation criteria, study designs tailored for clinical applications, and clinical validation. While direct evidence of clinical application was not found, the findings aim to guide future research and the implementation of digital image analysis in breast cancer diagnosis within clinical settings, potentially impacting the research community by advancing algorithmic applications in real clinical practice.

**Abstract:**

This systematic review aims to address the research gap in the performance of computational algorithms for the digital image analysis of HER2 images in clinical settings. While numerous studies have explored various aspects of these algorithms, there is a lack of comprehensive evaluation regarding their effectiveness in real-world clinical applications. We conducted a search of the Web of Science and PubMed databases for studies published from 31 December 2013 to 30 June 2024, focusing on performance effectiveness and components such as dataset size, diversity and source, ground truth, annotation, and validation methods. The study was registered with PROSPERO (CRD42024525404). Key questions guiding this review include the following: How effective are current computational algorithms at detecting HER2 status in digital images? What are the common validation methods and dataset characteristics used in these studies? Is there standardization of algorithm evaluations of clinical applications that can improve the clinical utility and reliability of computational tools for HER2 detection in digital image analysis? We identified 6833 publications, with 25 meeting the inclusion criteria. The accuracy rate with clinical datasets varied from 84.19% to 97.9%. The highest accuracy was achieved on the publicly available Warwick dataset at 98.8% in synthesized datasets. Only 12% of studies used separate datasets for external validation; 64% of studies used a combination of accuracy, precision, recall, and F1 as a set of performance measures. Despite the high accuracy rates reported in these studies, there is a notable absence of direct evidence supporting their clinical application. To facilitate the integration of these technologies into clinical practice, there is an urgent need to address real-world challenges and overreliance on internal validation. Standardizing study designs on real clinical datasets can enhance the reliability and clinical applicability of computational algorithms in improving the detection of HER2 cancer.

## 1. Introduction

Breast cancer (BC) remains the most common cancer among women globally [1]. Personalized approaches to diagnosing and treating BC underscore the need to precisely assess biomarkers such as human epidermal growth factor receptor 2 (HER2) [2]. HER2 expression is critical for predicting disease patterns and determining treatment strategies [3,4]. Since the late 1990s, immunohistochemical (IHC) screening of breast cancer patients for HER2 has been the standard practice due to the introduction of HER2-targeted therapies such as trastuzumab [5,6,7]. Trastuzumab reduces recurrence and mortality in early-stage HER2-positive BC by one-third and enhances chemotherapy outcomes in metastatic cases [3,8]. In recent studies, dual HER2-targeted therapy (pertuzumab and trastuzumab) further improved survival in metastatic BC [9].

HER2 status is primarily assessed by using IHC for protein expression and in situ hybridization (ISH) for gene amplification. ASCO/CAP guidelines recommend IHC screening, followed by ISH in equivocal cases, due to IHC’s high sensitivity, speed, and cost-effectiveness [2]. However, IHC has drawbacks, including manual assessment errors, inter-observer variability, and labor-intensive procedures caused by inconsistent staining and large slide areas [10,11,12,13,14,15,16]. Visual assessment led to notable inter-observer variability, particularly due to variations in staining across different laboratories and the requirement to estimate percentages within the tumor area. Approximately 20% of HER2 tests may be inaccurate due to various sources of variability (pre-analytic, analytic, or post-analytic) [17]. ASCO suggests that image analysis systems can improve HER2 test accuracy by providing quantitative measurements and enhancing the consistency of results [17].

Advancements in digital pathology and computer vision technologies are leading to the automation of IHC slide analysis, offering reproducible and objective interpretations of whole-slide images (WSIs) [18,19,20,21,22]. Automated scoring can mitigate the challenges of manual methods by reducing subjective bias and assisting pathologists in consistent scoring [23]. IHC quantification through image analysis, along with standardization and IHC automation, aims to improve marker reproducibility and foster further biomarker research [20,24,25]. Automated digital HER2 IHC analysis also addresses reproducibility issues, particularly with borderline HER2 values [26,27,28]. The College of American Pathologists is developing guidelines to improve IHC HER2 interpretation accuracy, precision, and reproducibility when interpreting IHC HER2 in BC, particularly where quantitative image analysis is used [29].

With the advent of digital pathology and image analysis, numerous algorithms have been developed for automating HER2 expression evaluation in experimental work. Despite these advancements, the integration of digital HER2 IHC analysis into national protocols has not occurred. While these algorithms demonstrate high performance, the feasibility of using and adopting the digital image analysis of HER2 in clinical practice, as well as its acceptability for clinical assessment, remain unclear. These uncertainties highlight existing gaps that require further exploration and resolution.

To the best of our knowledge, no previous systematic review has evaluated the effectiveness of HER2 automation classification algorithms in terms of clinical applicability. In light of this, we aimed to systematically assess the digital image analysis algorithms for HER2 in clinical settings, focusing on their performance criteria and key components to enhance the diagnosis of HER2 expression levels in breast cancer patients.

Key questions guiding this review include the following:

How effective are current computational algorithms at detecting HER2 status in digital images?

What are the common validation methods and dataset characteristics used in these studies?

Is there standardization for algorithm evaluations for clinical applications that can improve the clinical utility and reliability of computational tools for HER2 detection in digital image analysis?

## 2. Materials and Methods

### 2.1. Search Strategy

**Figure 1:**
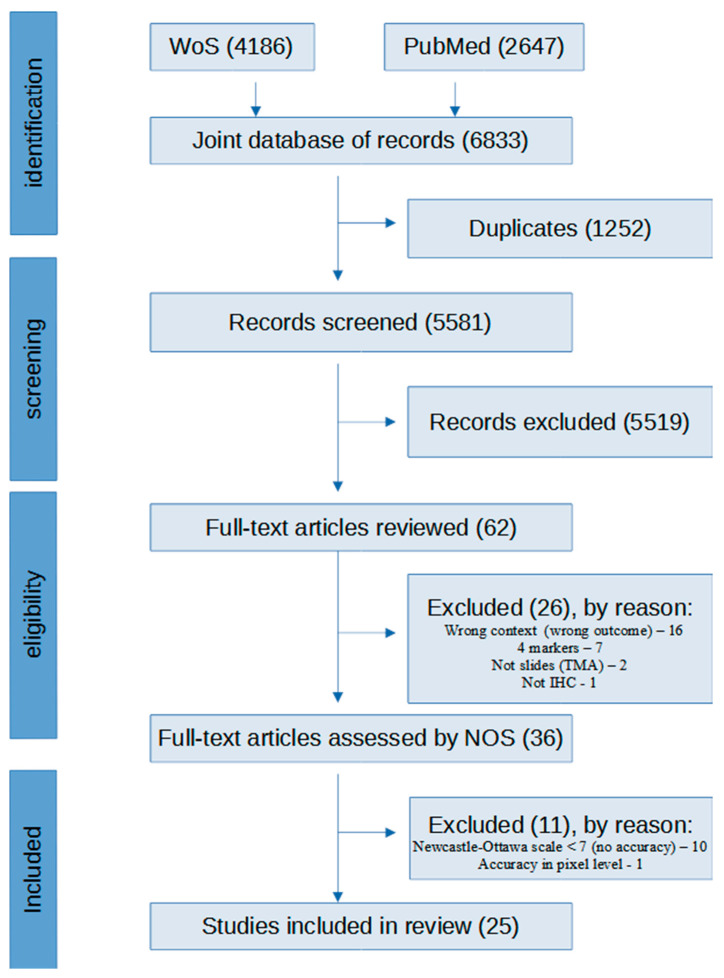
Study selection and eligibility criteria for systematic literature review. Abbreviations: TMA, tissue microarray; IHC, immunohistochemistry.

We conducted the search in two electronic literature databases: Web of Science and PubMed (latest search: 30 June 2024). The search strategy involved combinations of keywords related to breast cancer, HER2, immunohistochemistry, and digital image analysis algorithms. The full search strategy is provided in Table S1. By utilizing Medical Subject Headings (MeSH) and free-text keywords, we developed a search strategy based on the PEO conception, where P is population, E is exposure, and O is outcome. The time frame of the search was from 31 December 2013 to 30 July 2024. The search included articles in English only. To ensure the quality of our review, we followed the guidelines outlined by the Preferred Reporting Items for Systematic Reviews and Meta-Analyses (PRISMA) [30]. A flow diagram illustrating the process of study selection for this review is presented in Figure 1.

### 2.2. Study Selection

The inclusion and exclusion criteria for study selection were established by three reviewers (N.D., G.D., and N.G.) and are presented in Table 1.

Type of Participants: Studies involving breast cancer patients with known immunohistochemical markers.

Type of Exposure: Automatic analysis of IHC HER2 slides in digital WSI format.

Type of Outcome: The outcome focused on components of digital image analysis algorithms for HER2, facilitating their implementation in clinical settings. We considered factors essential for clinical use, including performance criteria (sensitivity, specificity, accuracy, precision, recall, F1 score, AUC-ROC), methods applied (algorithms, techniques, preprocessing steps), dataset characteristics (size, diversity, source), details on ground truth (method, level of annotation), and demonstration of clinical application (clinical validation, integration with clinical workflows).

Study Design: Original studies were eligible for inclusion. Reviews were excluded from the search.

### 2.3. Data Extraction and Quality Assessment

Recognizing the importance of specialized scales for assessing the diagnostic accuracy of methods, including those involving artificial intelligence, we aimed to evaluate the selected articles from a clinical use perspective. For this purpose, we assessed the risk of bias by using the Newcastle—Ottawa Scale (NOS), considering scores ≥7 as indicative of higher quality. This assessment was conducted independently by the two primary authors, G.D. and N.G. The quality of the studies was evaluated based on six evidence-based criteria covering selection, comparability, and outcome. In cases where there was a disparity in the scores assigned by the two reviewers for a particular study, the reviewers reached an agreement following discussion.

### 2.4. Data Synthesis and Analysis

In total, 25 publications were considered suitable for inclusion. Data extraction from the selected papers was conducted by two authors, G.D. and N.G., who resolved any discrepancies through joint interpretation. Subsequently, G.D. and N.G. drafted the initial version of this paper, taking into account all the relevant papers. The final version was produced based on feedback from all authors and scores were obtained from the Newcastle—Ottawa Scale. Then, we summarized the key findings on the components of digital image analysis algorithms for HER2, emphasizing their potential for implementation in clinical settings, and elaborated on the resulting conclusions. Data on performance evaluation criteria and elements of digital image analysis algorithms for IHC HER2 were compiled and presented using a narrative synthesis. The systematic review was registered in PROSPERO (CRD42024525404).

## 3. Results

A combined search of Web of Science and PubMed identified 6833 publications, which, after screening by using the online software Rayyan (https://www.rayyan.ai/) (accessed on 14 March 2024) [31] and applying the Newcastle—Ottawa Scale, resulted in 25 articles included in the review; further details are provided in Figure 1.

In Table 2, we compile basic information from the included articles, encompassing general details, performance evaluation criteria, and limitations on clinical applicability. Table 2 includes extracted data: (i) last name of the first author and year of publication; (ii) dataset used; (iii) number of cases; (iv) features; (v) criteria of evaluation; (vi) key findings; and (vii) limitations.

### 3.1. Criteria for Performance Evaluation

**Figure 2:**
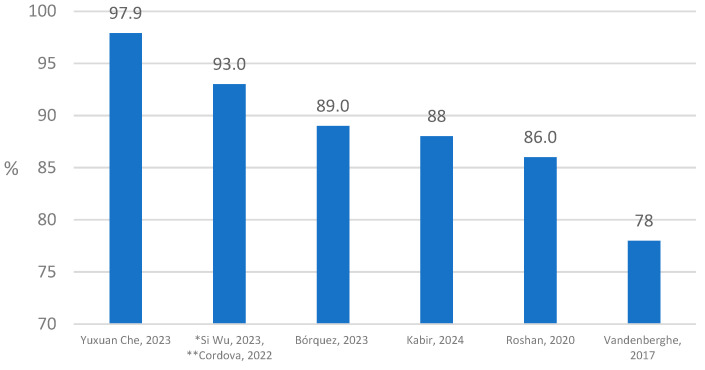
WSI level classifier accuracy for each class of HER2 (4 or 2) scores (n = 7) [26,32,33,40,47]. * Si Wu, 2023 [39]—for two classes of HER2 (0, 1+). ** Cordova, 2022 [41]—0.93 for the IHC + FISH model, 0.88 for the IHC model.

**Figure 3:**
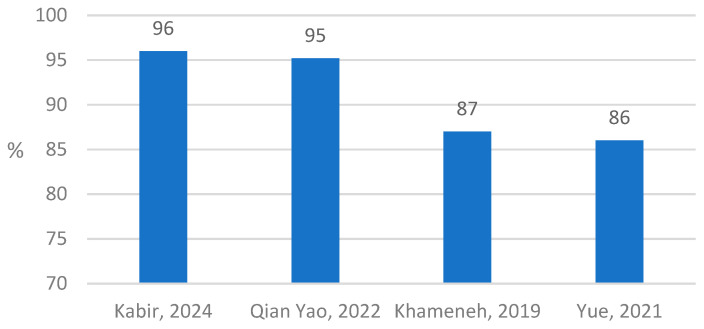
WSI level classifier accuracy of HER2 for three classes of scores (n = 4) [28,32,42,45].

**Figure 4:**
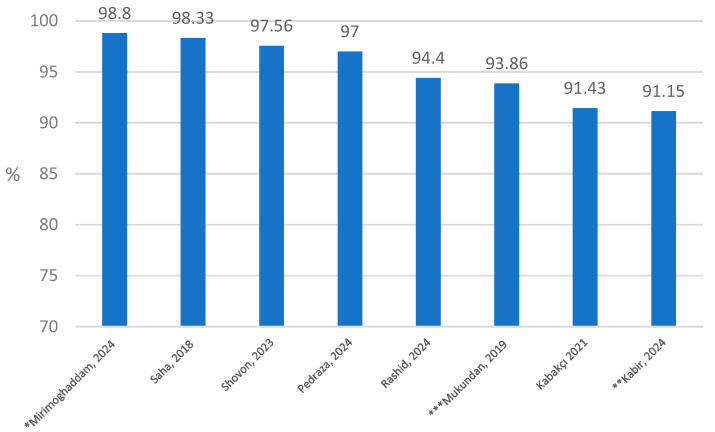
Patch score classifier accuracy for four class scores (n = 8) [36,50,51,52,54]. * Mirimoghaddam, 2024 [37]—98.8% in the augmented train + test Warwick dataset with the InceptionResNetV2 algorithm. ** Kabir, 2024 [32]—96.17% in DenseNet201. *** Mukundan, 2019 [34]—93.86% in logistic regression algorithm.

Among the articles that met the inclusion criteria, 21/25 authors included accuracy as one of the main performance evaluation criteria, while in three articles sensitivity and specificity were presented as evaluation criteria (Table 2). Accuracy was applied at the evaluation level of the WSI (nine studies) and patches (nine studies) (Figure 2, Figure 3 and Figure 4). Three articles provided accuracy on levels “Patch/WSI”, “Patch/Region of interest”, and “Foci of view”.

Additionally, the accuracy assessment was carried out for studies with all classes of HER2 (0, 1+, 2+, 3+) (13 studies), as well as for three classes where HER2 0 and 1+ were combined into one group (0/1+, 2+, 3+) (five studies). Two studies provided accuracy for HER2 low tumor, Wu et al. for (0, 1+), and Roshan et al. for (2+; both used clinical datasets. Out of 10 studies reporting WSI-level classifier accuracy, 7 provided accuracy metrics for each HER2 class, with the highest reported accuracy being 97.9% on a clinical dataset in the study of Che et al. [40] (Figure 2).

In four studies, the accuracy was provided for three classes of HER2. The highest accuracy in this group was achieved by Kabir et al. on a public dataset [32] (Figure 3); they also reported the accuracy for four classes of HER2 on the WSI and patch levels.

Of the 11 studies that reported patch-level classifier accuracy, 8 were assessed with four classes of HER2 and 4 with three classes.

Among the eight articles that assessed classifier accuracy at the patch level for four classes of HER2 scores, Mirimoghaddam et al. achieved the highest accuracy (98.8%) in fully augmented datasets (training and testing sets) based on the Warwick dataset with the InceptionResNetV2 algorithm [37]. This was the highest accuracy among all articles included in the review. The second-highest accuracy in this group was demonstrated by Saha at 98.33% [36] (Figure 4).

A series of works by Tewary et al. demonstrated accuracy at both the patch, image, and ROI levels for three classes: in 2021, the patch accuracy was 93% on images from a clinical dataset [43], 95% in 2022 using the Xception algorithm, and 96% with AutoIHCNet on the Warwick dataset [35].

In addition to using accuracy as the primary performance metric, 16 studies also included precision, recall, and the F1 score in their set of metrics (Table S2).

Pham et al. used a combination of precision, recall, and the F1 score for HER2 scoring in four classes on patch level, where F1 was 0.78, and the highest precision and recall in the testing set were in (2+) (0.909 and 0.937), and in (3+) (0.945 and 0.938) [38].

Five articles provided sensitivity and specificity as the main findings (Table 2): Palm et al. demonstrated sensitivity in the AI workflow for the IHC algorithm at 93.8%, 100% for the ISH algorithm, 96.1% for the specificity of the IHC algorithm, and 94.7% for the ISH algorithm [48]; Yim et al. achieved a sensitivity of 74.0% in image analysis compared with 72.1% in manual scoring and a specificity of 94.7% in image analysis in contrast with 96.2% in manual scoring [46]. Both authors used the HER2 4B5 algorithm in uPath Enterprise software (Roche Diagnostic International, Rotkreuz, Switzerland). In a study by Koopman et al. (2019), when comparing two platforms for DIA, the authors achieved sensitivities of 81.3% (Visiopharm) and 100% (HALO) and a specificity of 100% for both platforms [49]. Marcuzzo et al., in 2016, demonstrated a sensitivity of 100% and a specificity of 82% in their analysis of three classes of HER2 on the WSI level by using a specific software package, VISIA Imaging s.r.l. software (version 2.5.0.1, San Giovanni Valdarno, Italy) [53].

### 3.2. Characteristics of the Applied Methods

Out of 25 studies, 20 proposed algorithms (including two that used a microscope with AI) and five used algorithms from existing platforms. The vast majority of authors who developed their algorithms used deep learning—18 papers. Among the studies in which deep learning methods were used, two authors utilized an AI-assisted microscope with a HER2 scoring algorithm adhering to the 2018 ASCO/CAP guidelines: Yue (2021) [28] incorporated an AI-enhanced microscope featuring an augmented reality display and AI-driven computer unit for HER2 scoring based on membrane delineation and cell classification, while Wu et al. [39] employed a microscope equipped with a computer unit housing a HER2 scoring algorithm. An augmented reality module displayed the calculation results, with the algorithm leveraging membrane delineation, cell classification, and cancerous region segmentation.

ML was used as the sole method in two studies. Cordova et al. used a logistic regression-based supervised ML model to classify HER2 of IHC analysis and demonstrated one of the two highest accuracies in WSI level for all classes of HER2 scores (93%) [41]. In 2021, Tewary et al. utilized SVM with a Gaussian kernel for classification in an automated HER2 scoring approach. In their study, the AutoIHC Analyzer achieved an accuracy of 93%, and the accuracy of the ImmunoMembrane was 78% [44].

A few authors employed both machine and deep learning techniques [34,45,51]. Mukundan et al. used deep learning methods for cell region detection and classification of different types of cells (immune, stroma, tumor cells, and artifacts) and machine learning algorithms (logistic regression and SVM) for classifying HER2 [34]. Khamenen et al. used a combination of a superpixel-based SVM feature learning classifier for classifying epithelial and stromal regions and a convolutional neural network (CNN) for segmenting membrane regions in the WSI of breast cancer tissue samples [45].

Algorithms of currently available platforms were evaluated in five studies: a commercially available HER2 4B5 algorithm for automated scoring of HER2 protein expression levels in tissue samples developed by Roche Diagnostic International [46,48]. Koopman et al. compared HER2 image analysis between two independent platforms (the Visiopharm Integrator System (Denmark) and HALO (USA)) for inter-platform agreement, as well as with manual scores obtained from two pathologists (+ ISH in equivocal cases) [49]. Marcuzzo et al., in 2016, used VISIA Imaging software [53]. The publicly available ImmunoMembrane software (based on machine learning) was compared with manual assessment by an expert pathologist in the study of Roshan et al. [26], and in 2021, Tewary et al. compared HER2 scoring assessments from the proposed AutoIHC-Analyzer software (based on deep learning) and the ImmunoMembrane software with manual assessment by an expert pathologist [44].

### 3.3. Dataset Characteristics

Based on dataset utilization, studies can be categorized into three groups: those employing only clinical datasets, those using publicly available (and/or commercial) datasets, and those utilizing mixed datasets. Furthermore, we were interested in assessing the balance of HER2 score distributions across images and the number of WSIs employed. Among the 25 selected articles, clinical datasets were used as the only dataset by 11 authors [26,28,39,40,41,42,44,46,48,49,53] and in five studies as a component of mixed datasets along with the Warwick dataset [37,38,45,51,52].

In terms of the quantity of utilized images, the smallest clinical dataset was attributed to Yue et al. [28], comprising 50 WSIs. In this dataset, the authors deliberately omitted the HER2 0 group, with the exact number of removed images not specified. They retained 12 WSIs of HER2 (1+), 30 of HER2 (2+) (comprising 13 FISH-negative and 17 FISH-positive cases), and 8 WSIs of HER2 (3+). Conversely, the largest clinical dataset was reported by Yim [46], encompassing 555 patients in their primary research. Within this dataset, they documented 373 cases of HER2 (0), 61 cases of HER2 (1+), 46 cases of HER2 (2+) (including 29 SISH-positive instances), and 75 cases of HER2 (3+). Additionally, they included SISH results, with 451 cases recorded as SISH-negative and 104 as SISH-positive.

Among the 25 studies, 8 used publicly available datasets only, with the majority (6 studies) relying on the Warwick dataset [32,33,34,35,36,43]. The authors of these studies employed the Warwick dataset from 40 to 172 WSIs. Vanderberghe et al. utilized a dataset sourced either from AstraZeneca BioBank, Cambridge, UK (71 WSIs) or a commercial provider (Dako Denmark A/S, Glostrup, Denmark) [47]. Pedraza at al. (2024) used the AIDPATH dataset with 360 WSI, where 172 WSI came from Nottingham University Hospital (Warwick dataset) [50]. Shovon et al. employed the BCI dataset with 4870 image pairs of H&E and IHC [54].

A mixed dataset incorporating data from Warwick was used in five studies. Khamenen et al. utilized the Warwick dataset (79 WSIs for testing) and a clinical dataset (48 WSIs) [45]. Pham et al. employed a combination of three datasets, with 370 WSIs in total. The dataset comprised a clinical dataset (Erasme Hospital dataset, 270 WSIs), 50 WSIs from the Warwick dataset, and 50 WSIs from AIDPATH (Academia and Industry Collaboration for Digital Pathology) [38]. In the work of Mirimoghaddam et al., a combination of a clinical dataset (126 patients) and the Warwick dataset (the exact number of slides used in this study was not specified) was utilized [37]. Kabakçı et al. presented a clinical dataset combining the ITU-MED-1 (13 cases) and ITU-MED-2 (10 cases) with the Warwick dataset (79 WSIs) as a part of a mixed dataset [51].

Of the 20 studies that developed the algorithm approach, 11 utilized the Warwick dataset, including those that achieved the highest accuracy rates [32,36,37]. In all studies where a pre-existing algorithm was employed [26,46,48,49,53], a clinical dataset was utilized.

In 5 out of the 25 studies, the ground truth encompassed both components of HER2 diagnosis (IHC and ISH), which were applied across the entire datasets. In a study by Yim et al. (2019), the HER2 status of the dataset comprising 555 cases was confirmed through both IHC and SISH [46]. Koopman et al. (2019) also used both methods in the ground truth of 152 carcinomas [49]. Yao et al. (2022) confirmed, by ISH, the status of HER2 in a dataset of 228 cases [42]. In a study conducted by Gordova et al., the final diagnosis of 141 samples was supplemented with FISH (IHC + FISH) and utilized as training output [41]. This model exhibited enhanced performance in classifying HER2 photomicrographs compared to the IHC-only model. The authors proposed that incorporating FISH results as an additional dimension in the reference diagnosis could enhance the predictive capacity of the model, resulting in superior classification performance and more precise prediction of HER2 status. In a study by Roshan, all 60 samples were confirmed by ISH due to the study design (only equivocal cases in the dataset).

In addition, Palm et al. used evaluation of ISH of WSIs of all cases with an IHC HER2 consensus score of 1+ or above (55 WSIs out of 97 samples in a whole study cohort, which included 26 WSIs of 67 cases of primary tumors) [48].

Since deep learning in pathology requires a substantial volume of precisely annotated data, we were interested in the details of the process of labeling or annotating the images (at the WSI or patch level). Nineteen articles provided information regarding the labeling of the dataset, seven of which labeled the dataset as part of the original research [28,32,41,42,44,48,49]. Annotated datasets were mentioned in eight studies. The data annotation involved delineating the tumor region as the initial step in a subsequent HER2 assessment. Kabir et al. used annotation of patches on the presence of tumor cells with the aim of discarding patches with no tumor cells or very small regions with in-house constructed annotation software (1016 of 6641 patches were discarded) [32]. Pham et al. (2023) annotated tumor areas in 71 WSIs (Erasme and AIDPATH datasets) using Calopix software [38]. A large-scale annotation dataset (more than 20,000 image patches) was built in the work of Wu et al. for the forthcoming training of a cancerous region segmentation model (in addition to differentiating between in situ and invasive carcinomas, pathologists manually exclude in situ carcinomas) [39]. In a study by Che (2023), annotating cancer areas with concentrated and evenly distributed tumor cells of 23 WSIs from the total dataset (95 WSIs) was performed by two pathologists [40]. Yue et al. annotated patches from a training dataset (approximately 500 WSIs) in the preparation stage by identifying and delineating tumor areas by marking them as points on the images [28]. In the study of Khamenen et al., experts annotated features related to the classification of epithelial and stromal regions as the first step in automated scoring of HER2 and then features on tumor areas and cell membrane staining patterns of the dataset provided by Acibadem Hospital for training the segmentation model [45].

Vandenberghe et al. applied annotation with 18 biologically relevant features for each annotated cell, which was subsequently used for training classical machine learning models (SVM and random forests) [47]. Yim (2019) categorized the areas chosen by pathologists as annotated, referred to as “foci of view” (FOVs), since they were based on specific criteria on the intensity, thickness, and completeness of membrane staining observed in HER2 IHC-stained breast cancer specimens for further analysis [46].

In the majority of the studies included in the review, there was no strict balancing of the clinical dataset. A clinically balanced dataset was utilized by [39,44]. In most studies where the Warwick dataset was utilized, it was considered balanced [33,34,36,38,43].

Eight studies used cross-validation to evaluate the models: one study used 10-fold cross-validation [47], and four studies utilized a 5-fold cross-validation method [32,33,37,42,52]. Pham et al. used cross-validation with three different splits [38], and Mukundan mentioned a cross-validation set consisting of 30% of samples [34].

In 3 out of 25 studies, the authors indicated the use of an external dataset for validation. Khameneh et al. utilized a dataset from Acibadem Hospital to train their segmentation model and employed the Warwick dataset to test the model’s performance and validate the results [45]. Pham evaluated the proposed pipeline using the AIDPATH dataset as an external dataset [38]. Kabakçı also used the Warwick dataset for external validation purposes after training and testing on the ITU-MED datasets [51].

Pedraza utilized the AIDPATH dataset, which was obtained from three medical centers. The dataset was divided into three parts: 70% for training, 20% for validation, and 10% for a hold-out test set [50].

Data augmentation was mentioned in 13 of 25 studies. In the selected studies, six augmentation techniques were used (Table S3). Almost all authors resorted to geometric augmentation in combination with other methods [28,38,42], as well as the sole type of augmentation [32,33,35,40,43,50,51,52]. One study [37] used image generation techniques, such as the conditional generative adversarial network (CGAN) model, to create new synthetic images based on patterns learned from a dataset to increase the sample size. While image generation is a related concept that involves creating entirely new images from scratch, it is not typically considered a form of augmentation [55].

### 3.4. Aspects of Clinical Use Perspectives

Although components of clinical application such as clinical validation and integration with clinical workflows were considered significant, no evidence of these aspects was found in the selected articles. Nevertheless, other important aspects of these studies should be highlighted in terms of clinical adaptation. Specifically, intratumoral heterogeneity and low HER2 expression were noted in six studies [38,39,42,47,48,53].

Wu et al. discussed the impact of HER2 intratumoral heterogeneity on the interpretation of HER2 IHC scores [39]. The authors noted that HER2 heterogeneity can contribute to the poor consistency of HER2 interpretation, especially in cases with HER2-low tumors. The intratumoral heterogeneity of HER2 was evaluated across different interpretation approaches, revealing significant heterogeneity in HER2 (0) (ultra-low) of 28% and 86% for HER2 (1+) cases. Pathologist review showed high accuracy in cases with homogenous staining but decreased accuracy in cases with heterogeneity. The AI algorithm significantly improved the accuracy of identifying heterogeneity types (accuracy of 0.68 to 0.89), particularly when detecting scattered-type heterogeneity, where the improvement was notable from 0.49 to 0.79.

In the work of Pham et al., cases of boundary or heterogeneous slides were described in which pathologists had difficulty deciding on the final label for tumor areas [38]. In such cases, the authors suggest the utility of the proposed approach, which includes a spatial class map representation and tumor surface percentages for invasive cancer within the slide, as a useful tool to assist pathologists.

In a study by Vandenberghe et al., 12 discordant cases were reassessed independently after applying the deep learning-based image analysis algorithm [47]. Heterogeneity in HER2 staining was significantly greater in discordant cases than in concordant. The presence of HER2 staining heterogeneity was identified as a significant factor associated with disagreements between automated scoring and pathologist assessments, emphasizing the importance of careful assessment in challenging cases to ensure diagnostic accuracy. Additionally, the study highlighted that technical artifacts and poor tissue quality can contribute to heterogeneity in HER2 staining, further complicating diagnostic ambiguity.

Yao et al. also reported that discordant HER2 IHC results were mostly caused by high intratumoral heterogeneity (6 of 13), and the identification of HER2 staining heterogeneity was a major factor in the disparity between automated scoring and pathologists [42]. The authors found that staining variability within tumor cells, nonspecific cytoplasmic staining, and nonspecific staining in ductal carcinoma in situ were key factors contributing to misinterpretations.

In the study by Palm, it was observed that, among the HER2-low tumor group, the AI (utilizing the HER2 4B5 algorithm) showed moderate concordance with the established ground truth (Cohen’s κ 0.54) [48]. The AI identified more tumors as HER2-low than the ground truth, resulting in a 16% increase in the proportion of identified HER2-low tumors. Pathologists demonstrated substantial agreement when classifying HER2-low tumors, with slightly lower but still considerable concordance observed within the “AI-assisted Pathologists” subgroup.

In a study by Marcuzzo et al., in 164 WSIs, 15% were found to be heterogeneous cases, and the percentage of FISH-discordant cases was 17% vs. 2% in homogeneous samples. The authors noted that variations in staining may arise either from true biological differences, such as focal hyperexpression, or from artifacts related to sample handling, fixation, and processing.

## 4. Discussion

While direct evidence of the clinical application of the digital image analysis of HER2 is still lacking, several important points highlight why developing this field is crucial, particularly given the significance of HER2. Advancing research in this area aims to overcome key challenges in assessing outcomes for different types of cancer, including breast cancer. The importance of accurately evaluating HER2 expression levels in breast cancer cannot be overstated. HER2 overexpression has dramatic clinical implications for patients, as gene amplification or protein overexpression leads to excessive and uncontrolled proliferation, enhanced angiogenesis and oncogenesis, and dysregulated apoptosis [4,56]. Breast cancer can have up to 25–50 copies of the HER2 gene and up to 40–100-fold increases in HER2 protein, resulting in the expression of 2 million receptors on the surface of tumor cells [57]. HER2 overexpression occurs in 15–30% of cases of invasive breast cancer [2,58].

HER2-positive breast cancer is characterized by its aggressive nature, poor prognosis, reduced sensitivity to anthracycline-based chemotherapy, and better response to HER2-targeted therapies such as trastuzumab, lapatinib, and pertuzumab [59,60], which significantly improve disease-free and overall survival rates.

Pathologists currently face challenges related to the performance, accuracy, and objectivity of assessments. Visual analysis of IHC for HER2 in BC is a complex and time-consuming process that requires a high level of expertise, particularly in cases of heterogeneous staining [48]. Moreover, in situ hybridization is costly and not always available, limiting its use for screening HER2 status in BC. Therefore, improving HER2 diagnostics, which also includes the application of computational algorithms, is an anticipated process that is undergoing evolutionary translational phases.

This systematic review addresses the effectiveness of digital image analysis for the HER2 immunohistochemical marker in breast cancer within clinical settings, focusing on performance evaluation criteria and key components of the approach. Our research questions were: How effective are current computational algorithms at detecting HER2 status in digital images? What are the common validation methods and dataset characteristics used in these studies? Is there standardization of algorithm evaluations of clinical applications that can improve the clinical utility and reliability of computational tools for HER2 detection in digital image analysis?

In terms of the effectiveness of computational approaches, the current algorithms demonstrate high accuracy in detecting HER2 status, with reported accuracies ranging from 86% to 98% at the WSI level and even higher at the patch level. In this review, the highest performance criteria were achieved on a dataset that was fully synthesized by a CGAN-based model used for both training and testing [37]. However, when using clinical datasets, performance may decrease, indicating possible limitations in the generalizability of the algorithms. This is due to differences in clinical data characteristics, such as sample diversity and image quality. On a clinical dataset with original images under the same conditions, the authors achieved a significantly lower accuracy. The authors highlighted the importance of the quality of the synthesized images generated by the CGAN model, which closely resembled that of the original clinical images. Despite the significant accuracy rates achieved, these results may not fully translate to clinical settings due to variations in real-world data, indicating that while its effectiveness is established, its applicability in clinical practice remains uncertain.

In 88% of studies, validation was performed on the same dataset used for model training. Only three authors (12%) indicated validation on a different dataset (external validation), which may serve as a more reliable indicator of algorithm performance generalization. A lack of validation on an external dataset is common in various other domains of AI applications within the field of pathology [61]. In a recent study by Tafavvoghi et al. on applying DL publicly available datasets of BC hematoxylin and eosin WSI, it was found that only 28% of the studies utilized multiple datasets, and 14% employed an external validation set. The authors proposed that there is a possibility of overestimating the performance of other developed models [62]. A meta-analysis conducted by Liu et al. in 2019, which compared the diagnostic accuracy of deep learning algorithms in medical imaging with that of specialists, also highlighted the issue that many deep learning studies lack external validation (of the 82 studies included, 25 studies had out-of-sample external validation) and adequate reporting [63].

In the context of utilizing algorithms for the digital analysis of biomedical images, dataset requirements play a crucial role in ensuring the accuracy and generalizability of the proposed methods [64,65]. We incorporated in our study an analysis of the availability, size, representativeness, balance, labeling, and ground truth methodology within datasets. In general, the challenge with the availability and quality of the dataset is addressed by utilizing public datasets or applying different methods of augmentation.

Both publicly available and clinical datasets were employed in the studies. There is a positive trend of using clinical datasets in experimental works: 64% of studies employed clinical datasets as either the only type or as a component of mixed datasets. This is an encouraging sign for the practical application of computational algorithms for determining HER2 status in real-world clinical settings.

The Warwick dataset was used in 44% of the selected studies. This is a public dataset that was organized by the University of Warwick, the University of Nottingham, and the Academic–Industrial Collaboration for Digital Pathology (AIDPATH) consortium for the HER2 challenge contest workshop held in 2016 [23]. The Warwick dataset consists of 172 WSIs (both IHC and H&E-stained slides) from 86 cases of invasive BC, with 79 IHC WSIs scanned by a Hamamatsu NanoZoomer C9600 scanner. The ground truth was provided as the clinical reports by at least two specialist consultants from a tertiary referral center for breast pathology (Nottingham University Hospitals, NHS Trust) for 52 cases (training dataset). The testing dataset comprised 28 cases, consisting of IHC- and H&E-stained WSIs without ground truth information supplied or publicly shared.

Aside from the Warwick dataset, which represents HER2 IHC, there are existing publicly available datasets, such as ITU-MED-1 (consisting of 13 cases and 191 tissue images) and ITU-MED-2 (comprising 10 cases and 148 tissue images). These datasets include ground-truth labels for both training and test sets, offering a distribution of sample scores that is both balanced and unbalanced [51,66]. In general, using publicly available datasets addresses issues of accessibility, consistent labeling, and experimental repeatability [67]. However, in clinical practice, it may not fully represent real-world challenges, such as image quality and quantity.

In the studies included in the review, ground truth for breast cancer most commonly involved expert pathologist opinion (68%)—for instance, two pathologists (an expert pathologist and a senior resident) [28,32,49] or three pathologists [42]. Twenty percent of studies utilized ISH in the ground truth analysis of the entire dataset.

Expert labels are an essential requirement for implementing the digital analysis of biomedical images [68]. In our review, we differentiated the labeling and annotating procedures according to Langlotzet et al., 2018, where labeling is the assignment of a category to an entire image, and annotation provides information about a particular component of an image [64]. Datasets usually require substantial effort for high-quality annotation to delineate the tumor region and identify HER2 status features, but this is a requirement for the further application of algorithms [69].

The size of the dataset is an important factor influencing effectiveness [70]. In 48% of the studies in this review, data augmentation was employed, including the generation of new synthetic images. Data augmentation in digital image analysis is used to increase the volume of training data by creating new variations of images based on existing ones. According to Moreno (2020), data augmentation can improve the prediction accuracy in the 1–3% range [71].

The representativeness of the sample ensures that the analysis results will reflect the real characteristics of the population to which they will be applied. A study by Althnian et al. demonstrated that the overall effectiveness of classifiers is more dependent on the representativeness of the sample than its size [72]. In digital HER2 analysis, representativeness implies the distribution of HER2-positive and HER2-negative cases, reflecting the real patient population. With the accumulation of knowledge on the molecular biology of breast cancer, there is currently a trend toward identifying another group, the HER2-low group, due to its clinical significance [73]. A more balanced representation of classes helps improve the model’s performance in model training [74]. The generation of new synthetic images is a technique for addressing the imbalance between class samples by adjusting the distribution of classes to prevent the model from being biased toward the majority class [55].

Based on the analysis of available articles, there is currently no uniform standardization for evaluating algorithms for clinical use, which makes it difficult to improve the clinical utility and reliability of computational tools for HER2 detection in digital image analysis. Different studies use different sets of criteria to evaluate algorithms: 64% of studies used a combination of accuracy, precision, recall, and F1 as a set of performance measures [75,76]. The accuracy of the articles varied depending on the HER2 score level, with the highest accuracy observed in cases with clearly positive or negative HER2 values. In contrast, cases with a HER2 score of 2+ demonstrated only average accuracy. Attempts to interpret HER2 IHC in equivocal 2+ cases using digital image analysis are illustrated in studies [77,78]. Since a HER2 score of 2+ does not provide a definitive result, further testing with in situ hybridization, which is expensive and not always available, is required. Therefore, from a clinical practice perspective, achieving high accuracy in interpreting equivocal HER2 cases using digital image analysis is essential, particularly for the clinical implementation of these algorithms.

The studies use a variety of datasets and assessment levels, including three or four classes (0, 1+, 2+, 3+) and image analysis levels such as whole-slide images (WSIs) and patches. The diversity of methods and approaches leads to difficulties in comparing results and reduces confidence in the applicability of algorithms in real clinical practice. The need for standardization becomes obvious to improve the quality and reliability of diagnostics.

The studies primarily emphasized developing and assessing deep learning-based computational approaches for automatically scoring HER2 in breast cancer using WSIs. The articles predominantly focused on utilizing AI, particularly for describing the technical aspects of system development and evaluation for HER2 IHC performance, as well as for conducting comparative analyses with existing methods.

When the analysis includes the AI approach for segmentation and classification, there is a clear predominance in the utilization of algorithms based on deep learning, a subfield of machine learning [79]. This may be attributed to the fact that the search was initiated in 2013, coinciding with the active development of convolutional neural networks in various domains, including biomedical imaging, particularly in cancer detection [80,81,82]. Histopathological images constitute a large dataset with high-resolution and complex tissue structures [83]. Deep learning algorithms offer clear advantages over humans when handling vast amounts of data, processing intricate nondeterministic data, and conducting a thorough analysis of potential information in datasets. Deep learning directly extracts features from raw data and utilizes multiple hidden layers to generate output data [84,85]. Accordingly, deep learning has been actively applied in digital histopathology, which is characterized by the complexity and diversity of data within images [86,87].

In various studies, intratumoral heterogeneity is observed in around 40% of breast cancers, mostly occurring in HER2-low tumors and contributing to resistance to anti-HER2 therapy [73,88,89,90]. In the majority of published data from completed and ongoing clinical studies, HER2-low status is defined as IHC 1+ or 2+ combined with a negative ISH test result [91]. While there are insufficient data to designate HER2-low breast cancer as an individual disease subtype to date, it remains important for patient selection, as certain individuals may derive treatment benefits [92,93,94,95]. A poor response to neoadjuvant chemotherapy determines the clinical significance of HER2-low tumors [96], but at the same time, correlates with favorable survival in hormone-positive patients compared with HER2-zero patients [97].

In this review, one-quarter of the articles (24%) addressed additional questions pertinent to both clinical significance and relevance for HER2 digital analysis: intratumoral heterogeneity and low HER2 status. In the automated quantitative assessment of HER2, the research emphasizes the challenges associated with evaluating discordant cases, primarily stemming from the high heterogeneity of intratumoral staining in HER2 IHC [23,42,47]. Kabir reported that the lowest precision and recall were in the HER2 (1+) group due to its staining features and similarity to the HER2 (0) group, but the separation of these groups was noticed as important in terms of therapeutic benefits.

Based on the included articles, the effectiveness of digital image analysis for HER2 detection and its applicability in real-world settings can be enhanced through several strategies. First, incorporating larger and more diverse datasets from multiple centers is crucial for improving model performance [33,35,36,41,48]. Additionally, integrating additional biomarkers and data sources, such as clinical and genomic information, can significantly enhance the predictive power of the approach [33,38,41].

Improving the interpretability and explainability of models is essential for building trust among medical professionals [39]. Standardizing sample processing and staining will enhance the quality of samples for digital analysis [53], and ensuring high-quality immunohistochemistry (IHC) slides will lead to better outcomes with digital image analysis [49]. Furthermore, further validation with larger and more diverse clinical datasets is necessary to ensure the robustness of the methods [26,38,39,46].

There is also a need for longitudinal studies to evaluate the long-term effectiveness and reliability of AI-based image analysis tools in clinical settings [39]. Such studies can provide insights into how these tools impact patient outcomes over time and help identify any potential issues that might arise with prolonged use.

Integrating AI systems more seamlessly into clinical workflows, ensuring real-time feedback for pathologists, and addressing regulatory compliance and user-friendly interfaces are important areas for future research and development [28,39]. Additionally, investigating the effects of training set size and the depth of experience of the operator annotating the training set on model performance is essential for optimization [47]. These strategies collectively aim to refine digital image analysis techniques for improved HER2 detection and broader clinical applicability.

Our systematic review has certain limitations. One of the primary limitations of our study is the restricted access to bibliographic sources; we relied on only two bibliographic sources. Additionally, we did not utilize technical sources such as IEEE. The majority of IEEE publications were conference papers (CPAPER) and were excluded from our search during the screening stage.

A variety of study designs are noted among authors in this field. Studies include comparative analyses of different platforms, evaluations of the effectiveness of various segmentation and classification algorithms, and comparisons with manual methods. The absence of a unified approach to defining ground truth is acknowledged. This diversity likely offers ample opportunities for interpreting results but also poses a challenge to practical implementation due to the lack of standardization in evaluation methodologies. The absence of standardization in algorithm methodology and assessment may impact the comparability of results across different studies and the practical implementation of developed methods.

In nearly all of the articles described except for three, validation was conducted on datasets collected under identical conditions. From the perspective of potential practical applications, we consider this a significant limitation. Validating on a single dataset, part of which was already used for training, may result in an overestimation of the model’s true performance. This can lead to overfitting and an inaccurate assessment of the model’s ability to generalize to new data. Additionally, using the same dataset for both training and validation may obscure overfitting issues, thereby rendering the model less capable of generalizing to new data due to insufficient testing across diverse datasets.

The most significant limitation was the inability to apply the accuracy criteria achieved in the studies by the authors in practical settings, as information on the implementation of these criteria in practice is lacking.

While our study utilized the Newcastle—Ottawa Scale (NOS) to assess the quality of reviewed articles with a focus on clinical relevance, we acknowledge a key limitation: the lack of a dedicated methodology for appraising articles concerning the clinical application of AI approaches. This underscores the necessity for more refined and standardized criteria tailored specifically for evaluating AI articles within clinical contexts. One promising avenue is the potential utilization of QUADAS-AI, a framework awaiting further validation and real-world testing.

Thus, the evaluation criteria for algorithm effectiveness in the digital analysis of HER2 IHC images in breast cancer depend on the interaction of various factors. It is essential to recognize that the successful implementation of AI in digital pathology for a wide range of applications requires careful consideration of various factors to ensure accuracy, reliability, and clinical utility. Achieving high accuracy in the digital analysis of HER2 IHC images in breast cancer is crucial but not the sole criterion to be considered for future application in clinical practice under real-world conditions.

## 5. Conclusions

This systematic review highlights advances in digital HER2 analysis in breast cancer, emphasizing ongoing research efforts and improvements in accuracy. Although existing studies demonstrate promising results and explore key components for real-world implementation, such as the use of clinical datasets, further research is needed to integrate these methods into clinical workflows to ultimately enhance patient care.

Future research should address key issues such as developing robust external validation frameworks, incorporating diverse and representative clinical datasets, improving annotation and ground truth standards, and focusing on tumors with heterogeneity and HER2-low expression. By targeting these areas, researchers can contribute to the development of more reliable and clinically applicable computational tools for HER2 detection.

## Figures and Tables

**Table 1:** Inclusion and exclusion criteria for study selection.

Inclusion Criteria	Exclusion Criteria
Experimental or clinical studies on the use of AI in DIA of HER2 in patients with BC Availability of full-text studies Availability of criteria of performance evaluation (image, slide, patch levels) Availability of the components of DIA	Studies outside of the scope of the search Reviews (systematic, scoping) Studies with no criteria for performance evaluation

Abbreviations: AI, artificial intelligence; DIA, digital image analysis; HER2, human epidermal growth factor receptor 2; BC, breast cancer.

**Table 2:** The main characteristics of the included studies.

Author	Brief Presentation of HER2 Classifying Method	Dataset (Public/Clinical)	Total Number of Cases/Number of Cases in Each Class	Features	Criteria of Evaluation	Key Findings	Limitations
Kabir, 2024 [32]	Three key stages: tumor patch classifier, patch score classifier, and WSI-level score classifier.	Public (Warwick)	86 WSI (77 in final) /6641 patches **(0)** 9 **(1)** 11 **(2)** 36 **(3)** 21	DL models (DenseNet201, GoogleNet, MobileNet, Vision Transformer—ViTs).	Accuracy: ViTs: 92.6% (tumor patch classifier); patch score classifier: The RF: 91.15% (4 classes, patch), 88% (4 classes, WSI); 96% (3 classes, WSI); DenseNet201: 96.17%.	Annotated dataset (50 WSIs); internal validation; ground truth: 2 pathologists’ assessment.	No clinical dataset;no external validation.
Bórquez, 2023 [33]	Patch-level classification with different dropout rates and aggregation methods to classify tissue objects. Patch-level predictions were combined for classifying HER2 images at the tissue object level.	Public (Warwick)	52 WSI **(0)** 13 **(1)** 13 **(2)** 13 **(3)** 13	DL (Bayesian neural networks with Monte Carlo dropout).	Accuracy (4 classes, WS-tissue level): 0.89 on average.	Dataset, labeled previously; balanced dataset; Internal validation (5-fold CV); ground truth: pathologist’s assessment.	No clinical dataset; no external validation.
Mukundan, 2019 [34]	Characteristic curves for representing the % of staining, rotation-invariant uniform local binary pattern curves as texture descriptors, and a connectedness measure as a morphological feature of the staining patterns.	Public (Warwick)	52 WSI /4019 image patches **(0)** 13 **(1)** 13 **(2)** 13 **(3)** 13	DL for cell region detection and classification. ML (Logistic regression, SVM) for scoring.	Accuracy (4 classes, patch level): Average = 91% logistic regression algorithm: 93.86% SVM: 89%.	Dataset, labeled previously, balanced; ground truth: pathologist’s IHC assessment; internal validation (CV, 70%:30% images).	No clinical dataset; no external validation.
Tewary, 2022 [35]	2 CNN networks were compared with ImmunoMembrane.	Public (Warwick)	40 WSI of 3 classes (from 52 WSI with 13 cases for 4 classes (0, 1+, 2+, and 3+)).	Transfer learning (Xception); DL CNN (AutoIHCNet); ImmunoMembrane.	Accuracy (3 classes): Patch-based score: Xception—95% AutoIHCNet—96% ROI image-based score: Xception—97% AutoIHCNet—98% ImmunoMembrane—87%.	Dataset, labeled previously; ground truth: pathologist’s IHC assessment; internal validation (train-30 labeled images, test—10 WSIs).	No clinical dataset; no external validation.
Saha, 2018 [36]	Semantic segmentation of cell membrane and nucleus detection and scoring.	Public (Warwick) 188 for each score, i.e., 0, 1+, 2+, 3+	79 WSIs/752 core images **(0)** 188 core images **(1)** 188 **(2)** 188 **(3)** 188	DL (Her2net—LSTM recurrent network).	Accuracy (4 classes, patch level): 98.33%.	Dataset, labeled previously, balanced dataset; ground truth: pathologists assessment; internal validation (train: 51 WSIs; test: 28 WSIs).	No clinical dataset; no external validation.
Mirimoghaddam, 2024 [37]	GAN-based model was used for generating high-quality HER2 images to overcome the scarcity of HER2 images; 5 different types of classifiers were used for HER2 classification (MobilenetV2, InceptionV3, InceptionResNetV2, ViT, and Swin-T).	Mixed dataset (Warwick, clinical).	Clinical—126 patients: **(0)** 32 **(1)** 40 **(2)** 30 **(3)** 24	Transfer learning (HER2GAN).	Accuracy (4 classes, patch level, with InceptionResNetV2): 98.8% (Warwick, synthetic train + test sets); 90.5% (Warwick, original train + test sets); 85.71% (clinical dataset, original train + test sets) 92.13% (clinical dataset, synthetic train + test sets).	Labeled datasets; ground truth in a clinical dataset: pathologist’s assessment; internal validation (5-fold CV, 80%:20%).	No external validation; Best accuracy rates were achieved on a fully synthetized dataset based on Warwick.
Pham, 2023 [38]	An interpretable, weakly supervised constrained deep learning model for HER2 scoring.	Mixed dataset: Warwick, clinical (Erasme), and AIDPATH.	Clinical (270 WSIs)/Warwick (50 WSIs) **(0)** 49/13 **(1)** 76/12 **(2)** 109/12 **(3)** 36/13 AIDPATH (50 WSIs) 37 negative 6 equivocal 7 positive.	DL	(4 classes, patch level) F1 score: 0.78 Precision in the testing set (0) 0.822 (1+) 0.841 (2+) 0.909 (3+) 0.845 Recall in the testing set (0) 0.839 (1+) 0.905 (2+) 0.937 (3+) 0.938.	Clinical dataset (Erasme): labeled previously, annotated; AIDPATH datasets: labeled previously, annotated. Ground truth based on the clinical outcomes (negative, equivocal, positive). Erasme and AIDPATH were used to train the model to segment all tumor pixels. The patches are randomly split into 80%:10%: 10% for training, validation, and test set. Warwick: labeled previously as a part of the HER2 scoring contest training set.	No accuracy.
Si Wu, 2023 [39]	The authors conducted 2 rounds of HER2 0 and 1+ assessment. The first ring study (RS1) involved 15 pathologists interpreting 246 HER2 IHC sections via conventional microscopic examination. The second ring study (RS2—pathologist review): pathologists reassessed images with AI assistance using an AI microscope (by embedding an augmented reality module under the microscope eyepiece). The study aimed to improve the accuracy of HER2 0 and 1+ assessment and evaluate the role of AI in assessing low HER2 heterogeneity.	Clinical	246 cases **(0)** 120 **(1)** 126 **(2)** Out of scope **(3)** Out of scope (0) 120, (1+) 126.	DL (Microscope with AI).	Accuracy (2 classes (0, 1+), WSI level). RS1 (pathologists review) 0.80 RS2 (pathologists + microscope with AI) 0.93.	Balanced dataset; Annotation was likely conducted for detecting tumor areas; ground truth: pathologist’s IHC assessment; internal validation (validation conducted through a multi-institutional two-round ring study involving 15 pathologists with varying levels of experience).	No external validation.
Yuxuan Che, 2023 [40]	Binary classification of labeled patches (tumor patch/normal patch), WSI segmentation, scoring by integrated calculation of staining intensity, circumferential membrane staining pattern, and proportion of positive cells.	Clinical	95 WSI **(0)** 14 **(1)** 25 **(2)** 36 **(3)** 20	DL (ResNet)	Accuracy (4 classes) 73.49% (segmentation, patch level) 97.9% (scoring, WSIs).	Annotation of concentrated tumor areas (23 WSIs); ground truth: pathologist’s IHC assessment; 16 WSI for training, 79 for test.	No external validation.
Cordova, 2022 [41]	Classification of photomicrographs of HER2 using pathologists’ diagnoses (IHC only) vs. the final diagnosis (IHC + FISH) as training outputs, with applying of an explainability algorithm based on Shapley Additive exPlanations (SHAP) values to determine feature contributions (IHC only vs. IHC + FISH).	Clinical	131 patient samples and 10 controls (423 photographs) + 30 control samples (with and without tumor).	Supervised ML (logistic regression-based).	Accuracy (2 classes (IHC model and IHC + FISH model), WSI level): 0.88 (IHC model) 0.93 (IHC + FISH).	Dataset, labeled previously; ground truth: IHC+ ISH (previous reporting of pathologist’s diagnosis by IHC and IHC + FISH) 0.65:0.35 a ratio of training/testing sets.	No external validation.
Qian Yao, 2022 [42]	Predicting HER2 expression level in IHC and HER2 gene status in FISH analysis and comparing two models (GrayMax and its updates model, GrayMap + CNN).	Clinical	228 biopsy cases of IBC-NST with both IHC and FISH information **(0)** 5 **(1)** 21 cases (FISH: negative—19, positive—2) **(2)** 157 cases (FISH: negative—104, positive—53) **(3)** 45 cases (FISH positive—45).	DL (GrayMap+ CNN, GrayMax).	Accuracy (3 classes, WSI level): 95.20% (GrayMap + CNN) 84.19% (GrayMax).	Labeled dataset; ground truth: IHC and FISH. For IHC, manual assessment of 3 “blinded” pathologists (2 times after a 4-week washout); for ISH, 2 pathologists evaluated HER2/CEP17 the HER2 ratios of 20 tumor cells independently and blinded to IHC results; internal validation (5-fold CV).	No external validation.
Meng Yue, 2021 [28]	1st ring study: 33 pathologists from 6 hospitals read 50 HER2 WSIs through an online system. 2nd ring study: pathologists read HER2 slides using a conventional microscope. 3rd ring study: the pathologists used our AI microscope (Sunnyoptic ARM50) for assisted interpretation.	Clinical	50 WSIs consisted of 50% HER2-negative cases and 50% HER2-positive cases, with a total of 25 cases in each category. **(0)** Removed (to increase the efficiency of the experiment) **(1)** 12 (FISH negative cases) **(2)** 30 (FISH: negative—13, positive—17) **(3)** 8.	(AI)–assisted microscope Sunnyoptic ARM50: equipped with a conventional microscope and an augmented reality module.	For 3 classes, WSI level “AI”: accuracy κ = 0.86 [95% CI 0.84–0.89] “Pathologist Review” accuracy κ = 0.84 [95% CI 0.82–0.86].	Labeled and annotated dataset; ground truth: IHC consensus scores of 2 pathologists and a 3rd pathologist for discordant cases. Annotated (identifying and delineating tumor areas as points on the image patches), approx. 500 WSI from the training dataset.	No external validation.
Tewary, 2021 [43]	Transfer learning is applied using five pre-trained deep learning architectures (VGG16, VGG19, ResNet50, MobileNetV2, and NASNetMobile) with modified output layers for three-class classification. A statistical voting scheme using the mode operator is employed to combine the patch-based scores and generate the final image-based HER2 score.	Public (Warwick)	40 cases **(0/1)** 14 **(2)** 13 **(3)** 13	Transfer learning; DL	Accuracy (3 classes, patch and image level, VGG19) 0.91 in training; 0.93 in testing (100 epochs) patch-based scoring; 0.98 in image-based scoring.	Balanced, previously labeled dataset; ground truth: IHC pathologist’s assessment; internal validation (30 cases in training = 2130 image patches; 10 test cases =100 images).	Combined classes of HER2 (0/1); no clinical dataset; no external validation.
Tewary, 2021 * [44]	The approach AutoIHC-Analyzer and a publicly available open-source ImmunoMembrane software were compared with the scores of expert pathologists.	Clinical (from confusion matrix on page 5).	180 images **(0/1)** 60 **(2)** 70 **(3)** 50	DL (AutoIHC-Analyzer); Classifiers: SVM with Gaussian kernel); ML; ImmunoMembrane.	Accuracy (3 classes) 93%—AutoIHC-Analyzer: 78%—Immuno Membrane: Accuracy.	Labeled dataset; ground truth: IHC score provided by the clinical experts; internal validation (90 images for validation).	Combined classes of HER2 (0/1); no external validation.
Khameneh, 2019 ** [45]	The authors proposed an approach based on (1) Superpixel-based SVM classifies epithelial/stromal regions, (2) CNN segments membrane areas on epithelial regions, and (3) merged tiles evaluate slide scores. Experimental results compared with state-of-the-art handcraft and deep learning-based approaches.	Mixed: Warwick and clinical. Modified U-Net for classification.	total 127 WSIs Warwick dataset—79 WSIs. **(0/1)** 23 **(2)** 14 **(3)** 15 Clinical dataset (from Acibadem)—48 WSIs.	Modified U-Net for classification ML (SVM) for segmenting, classifying and quantifying. DL (CNN) for segmentation.	Accuracy (3 classes, WSI level) 0.87%—classification accuracy 0.9482%—segmentation accuracy.	Warwick dataset: labeled previously, ground truth: FISH and HER2 IHC (pathologist’s assessment); used for testing. Clinical dataset (from Acibadem): annotated (on tumor areas, cell membrane staining patterns, epithelial and stromal regions); ground truth: pathologist’s assessment; used for training.	Combined classes of HER2 (0/1)
Kwangil Yim, 2019 [46]	The results of the HER2 image analysis software (Companion Algorithm HER2 (4B5) image analysis software (Roche) compared with the manual scoring method and with HER2 SISH results (as the gold standard)). Previously, the authors found that at least 1000 tumor cells need to be examined in the most strongly stained areas (foci of view).	Clinical	555 patients in main research: SISH: (negative) 451, (positive) 104. **(0)** 373 **(1)** 61 **(2)** 46 (29 SISH-positive) **(3)** 75 32 HER2 2+ for preliminary test.	Companion Algorithm HER2 (4B5) image analysis software (Roche).	Accuracy (4 classes, foci of view level) 91.7%—manual scoring; 90.8%—image analysis.	Preliminary research resulted in using the approach of analyzing a certain area (40,457.64 μm^2^) until FOVs with at least 1000 tumor cells were assessed. Pathologists selected areas (“foci of view” (FOVs)) for further analysis. FOVs were chosen based on the following criteria: intense, thick, and complete membrane staining in the HER2 IHC-stained breast cancer specimens; ground truth: based on HER2 SISH.	No external validation.
Vandenberghe, 2017 [47]	Results of ConvNets for HER2 cell assessment were evaluated and compared to classical machine learning techniques (hand-crafted features + LSVM; hand-crafted features + RF).	Private dataset: from the AstraZeneca BioBank or acquired from a commercial provider (Dako Denmark A/S).	71 WSI Negative—43 Equivocal—17 Positive—11.	DL (ConvNets—Custom CNN).	Accuracy (four classes, WSI level) ConvNets 78% overall accuracy.	Annotated dataset (A total of 12,200 cells from a subset of 18 WSIs) was manually annotated by extracting 18 biologically relevant features (cell morphology, nuclear color, texture, and HER2 membrane staining), training classical machine learning models. The dataset was annotated using Definiens Developer XD for cell detection and feature extraction). Ground truth: manual annotation of cell features and HER2 scoring; internal validation (10-fold CV).	No external validation.
Palm, 2023 [48]	Results of groups (“pathologists” and “pathologists + AI”) were compared with AI results and a ground truth.	Clinical	a preliminary cohort: 495 newly diagnosed primary IBCs and their 30 metastatic BC (475 in total): **(0)** 181 **(1)** 156 **(2)** 87 **(3)** 51 a study cohort, 97 (all 30 metastatic tumors and their matched primaries and a further random selection of primary tumors from the preliminary cohort (67 primary tumors)). ISH on 55/97 samples of all cases with an IHC HER2 score of ≥1+ 26/67 from primary tumors with IHC 1+ or above were assessed by ISH.	HER2 4B5 algorithm in the uPath enterprise software (Roche Diagnostic International, Rotkreuz, Switzerland).	Sensitivity/specificity (slide level): 93.8%/96.1% for the IHC algorithm 100%/94.7% for the ISH algorithm.	Ground truth: consensus in the pathologists’ opinions. For IHC, a manual consensus score of three pathologists. For equivocal results of ISH (in HER2 2+), recounting the ISH signals of 20 cells by a second pathologist. As a result of testing the AI-IHC algorithm on the preliminary cohort, changes in incubation and counterstaining time of the automated slide stainer were applied. The adjusted protocol was used for a newly prepared study cohort.	No accuracy; no external validation.
Koopman, 2019 [49]	HER2 image analysis was compared between two independent platforms (Visiopharm Integrator System (Denmark) and HALO (USA) for inter-platform agreement, as well as with the manual score.	Clinical	152 Resection specimens of consecutive primary invasive breast carcinomas. 136 ISH Negative 16 ISH Positive **(0/1)** 114 **(2)** 23 **(3)** 12.	Visiopharm; HALO	Sensitivity/specificity (3 classes, slide level): Visiopharm: 81.3%/100% HALO: 100%/100%.	Ground truth: manual scoring by two independent pathologists and ISH in 2+ cases.	Combined classes of HER2 (0/1); no external validation.
Pedraza, 2024 [50]	Color transfer for data augmentation was employed on the initial dataset (DS1) to create a new dataset (DS2) with five classes: background, 0, 1, 2+, and 3+. Additionally, a separate dataset (DS3) was created with seven classes, including 1.5+ and 2.5+. The results from DS3 were then merged back into five classes for comparison. Multiple CNNs were applied for patch-wise grading of HER2.	AIDPATH	306 WSIs from 153 BC from 3 centers: 172 WSI from NHS (Warwick); 104 WSI from SESCAM; 30 WSI from SAS **(0)** 78 **(1)** 74 **(2)** 76 **(3)** 78	DL: five different CNNs (AlexNet, GoogleNet, VGG, ResNet-101, DenseNet-201).	Average accuracy = 97% for DenseNet-201 on DS2 (dataset 2–5 classes: background, 0, 1, 2, 3; balanced, augmented) **(0)** 0.95 **(1)** 0.94 **(2)** 0.96 **(3)** 0.98 Best accuracy (4 classes, WSIs)— ResNet-101 applied to DS3 dataset with 7 classes (dataset 3–7 classes: background, 0,1, 1.5, 2, 2.5, 3) **(0)** 0.968 **(1)** 0.954 **(1.5)** 0.975 **(2)** 0.974 **(2.5)** 0.986 **(3)** 0.988.	Previously labeled dataset; ground truth: at least 2 pathologists’ scoring; 70%:20%:10% WSI for training, validation, and as a hold-out test set.	No external validation; no clinical dataset.
Kabakçı 2021 [51]	Hybrid Cell Detection and Membrane Intensity Histogram Extraction methods were sequentially used for HER2 scoring, with testing on public and clinical datasets, and results were compared with ImmunoMembrane.	Mixed (clinical: ITU-MED-1, ITU-MED-2; Warwick).	ITU-MED-1: 13 cases/191 tissue images: (0) 41 (1) 42 (2) 52 (3) 56 ITU-MED-2: 10 cases/148 tissue images: (0) 24 (1) 18 (2) 49 (3) 57. Warwick—79 WSI	DL (LSTM); ML (k-Nearest Neighbors (kNN), Decision tree classifiers) for classification.	Accuracy (4 classes, patch-based) 91.43% ITU-MED-1, Best validation accuracy: 88.01% (LSTM), Best tissue-based scoring accuracy: 91.43% (Ensemble Boosted Trees); Compared with 74.07%. (ImmunoMembrane); ITU-MED-2, Best validation accuracy: 88.88% (LSTM), Best tissue-based scoring accuracy: 90.19% (Ensemble Boosted Trees, Ensemble Bagged Trees. Weighted kNN); Compared with 80.39% (ImmunoMembrane).	Ground truth: labeling by expert pathologists + FISH; The ITU-MED-1: 105 images for training, 86 for testing; the ITU-MED-2: 96 images for training, 52 for testing; Warwick: 51 WSI for training, 25 for testing.	
Rashid, 2024 [52]	A combination of a transfer learning model (ResNet50) for feature extraction, a metaheuristic optimizer (NSGA-II) for selecting the most relevant features, and a machine learning algorithm (SVM) for classification was applied and tested on two datasets.	Mixed (Warwick, clinical)	Warwick (HER2SC) 79WSI clinical—126 individuals (HER2GAN): **(0)** 32 **(1)** 40 **(2)** 30 **(3)** 24.	Transfer Learning Model (an ML strategy)—Resnet50; NSGA-II algorithm; SVM classifier.	Best accuracy (four classes, patch level) (Resnet50 + NSGA-II + SVM): 94.4% on HER2SC; 90.75% on HER2GAN.	5-fold CV, 80%:20%.	No external validation for either dataset.
Roshan, 2020 [26]	Digital image analysis using a free web application.	Clinical	**(2)** 60 samples/307 images.	ImmunoMembrane.	Accuracy (class “2+”, patch level) 86%.	Ground truth: manual IHC scoring by 2 pathologists and ISH.	No external validation.
Marcuzzo, 2016 [53]	Surgical samples and core biopsies were prepared for digital analysis by VISIA Imaging, and results were compared with FISH results.	Clinical	176 cases: 132 (75%) surgical specimens 44 (25%) biopsies. Negative (1+/0): 23 Equivocal (2+): 85 Positive (3+): 44 Inadequate: 24.	Specific software package: VISIA Imaging s.r.l. software (version 2.5.0.1, San Giovanni Valdarno, Italy).	Sensitivity/specificity (3 classes, WSI level) 100%/82%.	Comparison of results between types of specimen, staining distribution was done.	Combined classes of HER2 (0/1); no external validation.
Shovon, 2023 [54]	Several popular deep-learning architectures were employed for feature extraction and classification. Various activation functions were utilized to achieve better results. The classification results of the model trained on H&E and IHC images were compared.	Public	BCI dataset: 4870 image pairs with a resolution of 1024×1024 of H&E and IHC, equal number images of HER2 0, 1+, 2+, 3+.	DL, ML: DenseNet201- Xception-SIE (single instance evaluation) (with the best performance); InceptionResNetV2; VGG16; VGG19; ResNet101; ResNet152V2; EfficientNetB7; InceptionV3.	Best accuracy (4 classes, patch level) DenseNet201- Xception-SIE: 97.56% (on IHC data) 97.12% (on H&E data).	3896/977 images of H&E and IHC for training and validation.	No external validation; no clinical dataset.

* [44]—180 images with a resolution of 2240 × 1856 pixels. There were no data at the WSI or patch level. ** [45]—52 WSIs (used for training) from a total of 79 samples from the Warwick dataset were mentioned. There were no data on the remaining images. Abbreviations: HER2, human epidermal growth factor receptor 2; WSI, whole-slide image, ViTs, Vision Transformer; RF, Random Forest; CV, cross-validation; DL, Deep Learning; ML, Machine Learning; IHC, immunohistochemistry; SVM, support vector machine; CNN, convolutional neural network; ROI, region of interest; LSTM, long short-term memory; GAN, generative adversarial network (artificial intelligence algorithm); AIDPATH, Academia and Industry Collaboration for Digital Pathology (dataset); RS1, ring study; AI, artificial intelligence; FISH, fluorescence in situ hybridization; IBC-NST, invasive breast carcinoma of no special type; SISH, silver-enhanced in situ hybridization.

## Supplementary Materials

The following supporting information can be downloaded at https://www.mdpi.com/article/10.3390/cancers16152761/s1, Table S1: Web of Science and PubMed search, based on the Population-exposure-outcome criteria; Table S2: the characteristics of the performance evaluation criteria in the included studies; Table S3: data augmentation techniques used.
