# Supplementary material for: The Performance and Clinical Applicability of HER2 Digital Image Analysis in Breast Cancer: A Systematic Review
Source: Cancers (Basel). 2024 Aug 3;16(15):2761. doi: 10.3390/cancers16152761 (PMC11311684; doi:10.3390/cancers16152761)
Supplement: Supplementary file 1 [file cancers-16-02761-s001.zip › Supplemental Table S1-search_strategy.pdf]

Dunenova G et al., "The Performance and Clinical Applicability of HER2 Digital Image Analysis in Breast Cancer: A Systematic Review"

Supplemental Table S1. Web of Science and PubMed search, based on the Population-exposure-outcome criteria

Search number	Query
1	"Breast Neoplasms"[MeSH Terms] OR "breast"[MeSH Terms] OR "breast"[Title/Abstract]
2	"breast"[MeSH Terms] OR "breast"[Title/Abstract]
3	"neoplasm*"[Title/Abstract] OR "cancer"[Title/Abstract] OR "carcinoma*"[Title/Abstract]
4	#2 AND #3
5	"neoplasms, hormone dependent"[MeSH Terms]
6	#2 AND #5
7	"genes, erbB 2"[MeSH Terms] OR "receptor, erbB 2"[MeSH Terms] OR "erbB-2"[Title/Abstract] OR "HER2"[Title/Abstract] OR "human epidermal growth factor receptor-2"[Title/Abstract]
8	#2 AND #7
9	#1 OR #4 OR #6 OR #8
10	"Histology"[MeSH Terms] OR "Immunohistochemistry"[MeSH Terms] OR "Microscopy"[MeSH Terms] OR "Cytological Techniques"[MeSH Terms] OR "whole-slide imag*"[Title/Abstract] OR "WSI"[Title/Abstract] OR "digital scanner*"[Title/Abstract] OR "Immunohistochem*"[Title/Abstract] OR "Digital patholog*"[Title/Abstract]
11	"In Situ Hybridization, Fluorescence"[MeSH Terms] OR "FISH"[Title/Abstract]
12	#10 OR #11
13	("Algorithms"[MeSH Terms] OR "algorithm*"[Title/Abstract]) OR ("Machine Learning"[MeSH Terms] OR "Machine learning"[Title/Abstract]) OR ("Image Processing, Computer-Assisted"[MeSH Terms] OR "digital imag*"[Title/Abstract] OR "Picture Archiving and Communications System*"[Title/Abstract] OR "image process*"[Title/Abstract]) OR ("Artificial Intelligence"[MeSH Terms] OR "Artificial intelligence"[Title/Abstract] OR "AI"[Title/Abstract]) OR ("Deep learning"[MeSH Terms] OR "Deep learning"[Title/Abstract])
14	("Workflow"[MeSH Terms] OR "workflow"[Title/Abstract]) OR "digitization"[Title/Abstract] OR "automation"[Title/Abstract] OR ("Digital Technology"[MeSH Terms] OR "Digital Technolog*"[Title/Abstract])
15	("Medical Informatics Applications"[MeSH Terms] OR "Big Data"[MeSH Terms] OR "big data"[Title/Abstract]) OR ("Datasets as Topic"[MeSH Terms] OR "Information Storage and Retrieval"[MeSH Terms] OR "dataset*"[Title/Abstract] OR "digital repositor*"[Title/Abstract]) OR ("Decision Support Techniques"[MeSH Terms] OR "decision support*"[Title/Abstract])
16	#13 OR #14 OR #15
17	#9 AND #12 AND #16
18	"2013/12/31"[Date - Publication] : "2024/06/30"[Date - Publication]
19	#17 AND #18
20	"Systematic review"[Publication Type] OR "review"[Publication Type] OR "meta analysis"[Publication Type] OR "comment"[Title/Abstract] OR "editorial"[Title/Abstract] OR "letter"[Title/Abstract] OR "retracted publication"[Title/Abstract] OR "retraction*"[Title/Abstract]
21	#19 NOT #20
22	English[Language]
23	#21 AND #22
