# Supplementary material for: The Performance and Clinical Applicability of HER2 Digital Image Analysis in Breast Cancer: A Systematic Review
Source: Cancers (Basel). 2024 Aug 3;16(15):2761. doi: 10.3390/cancers16152761 (PMC11311684; doi:10.3390/cancers16152761)
Supplement: Supplementary file 1 [file cancers-16-02761-s001.zip › Supplemental Table S2-Evaluation_crit.pdf]

Dunenova G et al., "The Performance and Clinical Applicability of HER2 Digital Image Analysis in Breast Cancer: A Systematic Review"

Supplemental Table S2. Characteristics of the performance evaluation criteria in the included studies

**Author**	**Brief presentation of research**	**Dataset Cases/images**<br><br>**Method/Classifier**	**Accuracy/ Sensitivity/specificity**	**Precision**	**Recall**	**F1 Score**	**AUC-ROC**
Kabir, 2024<br><br>[36]	Authors proposed a deep learning-based automatic framework for the automatic detection of HER2 score from WSI, consisting of three key stages: tumor patch classifier, patch score classifier and WSI-level score classifier.	University of Warwick HER2 challenge contest.<br><br>86 WSI (77 in final) (0)9, (1+)11, (2+)36, (3+)21.<br><br>/6641 patches<br><br>DenseNet201, GoogleNet, MobileNet –CNN (Deep learning)<br><br>Vision Transformer - (ViTs)	For 4 classes on patch level:<br>**The vision transformer-based model:** Tumor patch classifier - accuracy: 92.6%<br><br>**The random forest classifier:**<br>Patch score accuracy: 91.15%,<br>4 class accuracy (WSI): 88%,<br>3 class accuracy (WSI): 96% (Tested on 77 WSI)<br><br>DenseNet201 accuracy for patch score classification: 96.17%	(0) 0.75<br>(1+) 0.64<br>(2+) 0.92<br>(3+) 1	(0) 0.67<br>(1+) 0.64<br>(2+) 0.97<br>(3+) 0.95	(0) 0.71<br>(1+) 0.64<br>(2+) 0.95<br>(3+) 0.98	Not available
Bórquez, 2023<br><br>[37]	Authors proposed a method for classifying HER2 images into 4 categories (0, 1+, 2+, 3+) using Bayesian deep	University of Warwick HER2 challenge contests training set only:	for 4 classes classification on a whole slide tissue-level:	0.81	0.74	Not available	Not available

	learning techniques and uncertainty estimation measures.<br>The method included patch-level classification with different dropout rates and aggregation methods to classify tissue objects. Patch-level predictions were combined for classifying HER2 images at the tissue object level.	52 images (equal number of 0, 1+, 2+, 3+). Was divided into training and testing sets with a ratio of 80:20.<br><br>Bayesian neural networks (Deep learning) with Monte Carlo dropout	0.89 on average				
Mukundan,<br>2019<br><br>[38]	Authors uses characteristic curves for representing the percentage of staining, rotation-invariant uniform local binary pattern curves as texture descriptors, and a connectedness measure as a morphological feature of the staining patterns.	University of Warwick HER2 challenge contest.<br><br>52 WSI (0) 13, (1+) 13, (2+) 13, (3+) 13.<br>/4019 image patches<br><br>Deep learning for cell regions detection and classification.<br>Machine learning (Logistic regression, SVM) for scoring.	For 4 classes, patch level:<br>Average - 91%<br><br>logistic regression algorithm:<br>accuracy 93.86%<br><br>support vector machine algorithm 89%	logistic regression algorithm:<br><br>precision<br>(0) 0.86<br>(1+) 0.94<br>(2+) 0.97<br>(3+) 0.98	logistic regression algorithm:<br><br>Recall<br>(0) 0.96<br>(1+) 0.81<br>(2+) 0.96<br>(3+) 1.0	logistic regression algorithm:<br><br>F1-Score (0) 0.91<br>(1+) 0.87<br>(2+) 0.96<br>(3+) 0.99	Not available
Tewary,<br>2022<br><br>[39]	2 CNN networks were compared with ImmunoMembrane	University of Warwick HER2 challenge contest.<br><br>40 WSI from 52 with 13 cases for four classes (0, 1+, 2+ and 3+).	For 3 classes.<br><br>Patch-based score:<br>Xception - 95%<br>AutoIHCNet - 96%	AutoIHCNet Image based scoring with statistical voting (100 epochs)<br><br>(0/1+) 0.98<br>(2+) 0.97	AutoIHCNet Image based scoring with statistical voting (100 epochs)<br><br>(0/1+) 1.00	AutoIHCNet Image based scoring with statistical voting (100 epochs)<br><br>(0/1+) 0.99<br>(2+) 0.97	Not available

		Grouped into 3 classes: (0/1+) neg, (2+) equiv., (3+) pos.<br><br>/2400 patches from 300 training ROI images.<br><br>a transfer learning framework - the Xception<br><br>AutoIHCNet<br>Deep learning	ROI image-based score:<br><br>Xception - 97%<br>AutoIHCNet - 98%<br>ImmunoMembrane - 87%	(3+) 1.00<br><br>Xception – I based scoring with statistical voting (75 epochs):<br>(0/1+) 0.95<br>(2+) 0.97<br>(3+) 1.00	(2+) 0.97<br>(3+) 0.97<br><br>Xception – I based scoring with statistical voting (75 epochs):<br>(0/1+) 1.00<br>(2+) 0.93<br>(3+) 0.97	(3+) 0.97<br><br>Xception – I based scoring with statistical voting (75 epochs):<br>(0/1+) 0.98<br>(2+) 0.95<br>(3+) 0.98	
Saha, 2018<br><br>[32]	Authors proposed and tested Her2Net - a fully deep learning-based semantic segmentation of cell membrane and nucleus detection, segmentation and scoring for IHC HER2 images.	University of Warwick HER2 challenge contest.<br><br>79 WSIs: training (51 WSIs) and testing (28 WSIs)<br>/752 core images<br><br>188 for each score, i.e., 0, 1+, 2+, 3+<br><br>Her2net—LSTM recurrent network, cell membrane and nuclei detection<br><br>Deep learning	For 4 classes, patch level:<br><br>98.33%	96.64%	96.79%	96.71%	Not available

Mirimoghaddam, 2024<br><br>[40]	Authors used a GAN-based model for generating high-quality HER2 images with the aim of overcome the scarcity of HER2 images and evaluated the identifying and classifying HER2 levels with 5 different types of classifiers - transfer learning models: MobilenetV2, InceptionV3, InceptionResNetV2, ViT, and Swin-T.	1 dataset - University of Warwick HER2 challenge contest.<br><br>2nd dataset- clinical: 126 patients: (0) 32; (1+) 40; (2+) 30; (3+) 24.<br><br>Deep learning (Transfer learning models)	Best performances for 4 classes, patch level<br><br>**Warwick dataset (WSI) train+test:**<br>(Original+ Original) InceptionResNetV2 - 90.5%<br><br>(Augment+ Augment) InceptionResNetV2 - 98.8%<br><br>(Augment+ Original) InceptionResNetV2 - 89.8%<br><br>(Combine (A + O)) InceptionResNetV2 - 94.2%<br><br>**Clinical dataset train+test:**<br>(Original+ Original) InceptionResNetV2 - 85.71%<br><br>(Augment+ Augment) InceptionV3 - 92.13%<br><br>(Augment+ Original) InceptionResNetV2 - 86.46%	**Warwick dataset (WSI) train+test:**<br>(Original+ Original) MobileNetV2, InceptionResNetV2 - 90.9%<br><br>(Augment+ Augment) InceptionResNetV2 - 98.8%<br><br>(Augment+ Original) ViT -90%<br><br>(Combine (A + O)) InceptionResNetV2 - 94.3%<br><br>**Clinical dataset train+test:**<br>(Original+ Original) InceptionResNetV2 - 87%<br><br>(Augment+ Augment) InceptionV3 - 93.7%<br><br>(Augment+ Original)	**Warwick dataset (WSI) train+test:**<br>(Original+ Original) InceptionResNetV2 - 90.5%<br><br>(Augment+ Augment) InceptionResNetV2 - 98.8%<br><br>(Augment+ Original) InceptionV3, InceptionResNetV2 - 89.1%<br><br>(Combine (A + O)) InceptionResNetV2 - 94.2%<br><br>**Clinical dataset train+test:**<br>(Original+ Original) InceptionResNetV2 - 86.5%<br><br>(Augment+ Augment)	**Warwick dataset (WSI) train+test:**<br>(Original+ Original) InceptionResNetV2 - 90.7%<br><br>(Augment+ Augment) InceptionResNetV2 - 98.8%<br><br>(Augment+ Original) InceptionResNetV2 - 89.2%<br><br>(Combine (A + O)) InceptionResNetV2 - 94.2%<br><br>**Clinical dataset train+test:**<br>(Original+ Original) InceptionResNetV2 - 86.75%<br><br>(Augment+ Augment) InceptionV3 - 92.9%<br><br>(Augment+ Original) InceptionResNetV2 - 86.4%	**Warwick dataset (WSI) train+test:**<br>(Original+ Original) InceptionResNetV2 - 93.95%<br><br>(Augment + Augment) InceptionResNetV2 - 99.8%<br><br>(Augment + Original) InceptionResNetV2 - 92.81%<br><br>(Combine (A + O)) InceptionV3 - 96.02%<br><br>**Clinical dataset**

			(Combine (A + O)) InceptionV3 - 90.75%<br><br>The InceptionV3 and InceptionResNetV2 models achieved a highest accuracy in both datasets, clinical dataset demonstrated less accuracy	InceptionResNetV2 - 86.4%<br><br>(Combine (A + O)) InceptionV3 - 91.63%	InceptionV3 - 92.13%<br><br>(Augment+ Original) InceptionResNetV 2 - 86.4%<br><br>(Combine (A + O)) InceptionV3 - 90.75%	(Combine (A + O)) InceptionV3 - 91.18%	**train+test:** (Original+ Original) Inception ResNetV2 - 88.81%<br><br>(Augment + Augment) Inception ResNetV2 - 94.56%<br><br>(Augment + Original) Inception ResNetV2 - 89.55%<br><br>(Combine (A + O)) Inception V3 - 93.68%
Pham, 2023<br><br>[41]	presented an interpretable weakly supervised constrained deep learning model for HER2 scoring.	370 (WSI) from 3 database:<br><br>270 WSI - clinical dataset,	Not available	Testing set<br>(0) .822<br>(1+) .841<br>(2+) .909<br>(3+) .845	Testing set<br>(0) .839<br>(1+) .905<br>(2+) .937<br>(3+) .938	0.78	Not available

		50 WSI from University of Warwick HER2 challenge contest,<br><br>50 WSI AIDPATH (37 negative, 7 positive, and 6 equivocal slides)<br><br>Deep learning					
Si Wu, 2023 [42]	The authors conducted 2 rounds of HER2 0 and 1+ assessment. In the first ring study (RS1) involved 15 pathologists interpreted 246 HER2 IHC sections via conventional microscopic examination. The second ring study (RS2-pathologist-review) - pathologists reassessed images with AI assistance using an AI microscope (by embedding an augmented reality module under the microscope eyepiece). The study aimed to improve the accuracy of HER2 0 and 1+ assessment and evaluate the role of AI in	Clinical:<br><br>246 cases (0) 120, (1+) 126<br><br>Microscope with AI Deep learning	2 classes (0, 1+), WSI level.<br><br>RS1 (Pathologists review) 0.80<br><br>RS2 (Pathologists + Microscope with AI) 0.93	RS1<br>(0) 0.76<br>(1+) N/m<br><br>RS2- pathologist-review<br>(0) 0.90<br>(1+) Not available	RS1<br>(0) Not available<br>(1+) 0.70<br><br>RS2- pathologist-review<br>(0) Not available<br>(1+) 0.90	RS1<br>0) 0.82<br>(1+) 0.78<br><br>RS2- pathologist-review<br>(0) 0.93<br>(1+) 0.93	Not available

	assessing low HER2 heterogeneity.						
Yuxuan Che, 2023<br><br>[43]	Authors proposed method based on a deep learning network (ResNet) for binary classification of labeled patches (tumor patch/normal patch), WSI segmentation, scoring by integrated calculation of staining intensity, circumferential membrane staining pattern, and proportion of positive cells.	Clinical dataset:<br>95 WSI: (0) 14, (1+) 25, (2+) 36, (3+) 20<br><br>16 WSIs for training, 79 for evaluation (test)<br><br>ResNet<br><br>Deep learning	For 4 classes, patch-level (segmentation accuracy):<br>73.49%<br><br>Slide-level (scoring Accuracy):<br>97.9%	segmentation precision - Patch-level:<br>95.77%	Segmentation recall - Patch-level:<br>73.38%	F1 for scoring - Patch-level:<br><br>83.09%	cell segmentation model's performance<br>0.983
Cordova, 2022<br><br>[44]	Authors trained a logistic regression-based supervised ML model for the classification with HER2 photomicrographs with using pathologists' diagnoses (IHC only) vs. the final diagnosis complemented with FISH (IHC + FISH) as training outputs.	Clinical dataset:<br>131 patient samples and 10 controls - 423 photographs<br><br>explainability algorithm based on Shapley Additive exPlanations (SHAP) values<br><br>Supervised machine learning	For 2 classes (IHC model and IHC + FISH model)<br>WSI level<br><br>for IHC model<br>0.88<br><br>for IHC + FISH<br>0.93	for IHC model<br>0.89<br><br>for IHC + FISH<br>1.00	for IHC model<br>0.43<br><br>for IHC + FISH<br>0.55		for IHC model<br>0.81<br><br>for IHC model<br>0.94
Qian Yao, 2022	Authors proposed GrayMap + CNN model	Clinical dataset:	For 3 classes, WSI level	Not available	Not available		Not available

[45]	for predicting HER2 expression level in IHC and HER2 gene status in FISH analysis and compared the performance of the GrayMap + CNN model with the GrayMax model using a 5-fold cross-validation method.	228 biopsy cases of IBC-NST with both IHC and FISH: (0) 5, (1+) 21 (with FISH neg 19, posit 2), (2+) 157 (2ith Fish negative - 104, positive – 53), (3+) 45 (Fish positive – 45)<br><br>GrayMap+ CNN<br><br>Deep learning	GrayMap + CNN 95.20%<br><br>GrayMax 84.19%			GrayMap + CNN 86.04%<br><br>GrayMax 66.54%	
Meng Yue, 2021<br><br>[28]	1st ring study - 33 pathologists from 6 hospitals, read 50 HER2 WSIs through an online system.<br>2nd ring study – pathologists read HER2 slides using a conventional microscope.<br>3rd ring study - the pathologists used our AI microscope (Sunnyoptic ARM50) for assisted interpretation.	Clinical dataset:<br>50 WSIs: (0) removed, (1+) 12, (2+) 30 (13 FISH-negative, 17 FISH-positive), (3+) 8.<br><br>(AI)–assisted microscope equipped with a conventional microscope and an augmented reality module	For 3 classes, WSI level<br><br>"AI": accuracy κ = 0.86 [95% CI 0.84–0.89]<br><br>"Pathologist Review" accuracy κ = 0.84 [95% CI 0.82–0.86]	Not available	Not available	Not available	Not available
Tewary, 2021<br><br>[30]	Transfer learning is applied using five pretrained deep learning architectures (VGG16, VGG19, ResNet50, MobileNetV2, and NASNetMobile)	Warwick dataset<br>40 cases: (0/1+) 14, (2+) 13, (3+) 13.<br><br>Transfer learning with modified output layers by removing the last fully	For 3 classes, patch and image level.<br><br>VGG19 0.91 (in training),	VGG19: patch-based scoring/image-based scoring:<br><br>Precision<br>(0/1+) 0.93/0.98	Recall:<br>(0/1+) 0.93/1.00<br>(2+) 0.92/ 0.97<br>(3+) 0.92/0.97	F1score:<br>(0/1+) 0.93/0.99<br>(2+) 0.89 /0.97<br>(3+) 0.96 /0.98	Not available

	with modified output layers for 3-class classification. A statistical voting scheme using the Mode operator is employed to combine the patch-based scores and generate the final image-based HER2 score.	connected layer and collective voting scheme.<br><br>VGG19<br>Deep learning	Testing (100 Epochs) patch-based scoring - 0.93,<br><br>image-based scoring - 0.98	(2+) 0.86/ 0.97<br>(3+) 1.00/1.00			
Tewary, 2021<br><br>*[31]	Authors compared their approach for automated cell membrane extraction followed by HER2 molecular expression assessment (AutoIHC-Analyzer) and publicly available open source ImmunoMembrane software with the scores of expert pathologist.	Clinical dataset:<br><br>180 images:<br>(0/1+) 60, (2+) 70, (3+) 50 (from confusion matrix on page 5)<br><br>Classifiers - SVM with Gaussian kernel<br><br>Machine learning	For 3 classes<br><br>AutoIHC-Analyzer: Accuracy 93%	(0/1+): 0.93<br>(2+): 0.89<br>(3+): 1.00	0.87<br>0.94<br>1.00	0.87<br>0,92<br>1.00	Not available
			Immuno Membrane: Accuracy 78%	(0/1+): 0.65<br>(2+): 0.89<br>(3+): 0.89	0.93<br>0.49<br>1.00	0,77<br>0,63<br>0,94	
Khameneh, 2019<br><br>**[46]	Authors proposed an approach based: 1) Superpixel-based SVM classifies epithelial/stromal regions. 2) CNN segments membrane areas on epithelial regions. 3) Merged tiles evaluate slide scores.	127 WSIs:<br><br>79 WSI –University of Warwick HER2 challenge contest,<br>48 WSI - clinical - Acıbadem hospital dataset for training	For 3 classes, WSI level.<br><br>Classification accuracy 0.87%<br><br>segmentation accuracy 0.9482%	Not available	Not available	Not available	Not available

	Experimental results compared with state-of-the-art handcraft and deep learning-based approaches.	Warwick dataset – 52* WSI for testing: (0/1+) 23, (2+) 14, (3+) 15.<br><br>modified U-Net for classification<br><br>Machine learning (SVM) for segmenting, classifying and quantifying.<br>Deep learning (CNN) for segmentation.					
Kwangil Yim, 2019<br><br>[47]	Authors compared the results of the HER2 image analysis software (Companion Algorithm HER2 (4B5) image analysis software (Roche) to the results of the HER2 manual scoring method and to HER2 SISH results (as the gold standard) in 555 breast cancer patients.<br><br>Previously authors found that at least 1000 tumor cells need to be examined in the most strongly stained areas (foci of view).	Clinical dataset:<br>32 HER2 2+ for preliminary test (for considering the number of cells in the foci of view)<br><br>555 patients in main research:<br>(0) 373, (1+) 61, (2+) 46 (29 SISH positive), (3+) 75.<br><br>SISH: (negative) 451, (positive) 104.<br><br>Companion Algorithm HER2 (4B5) image analysis software (Roche)	For 4 classes, Foci of view level.<br><br>manual scoring - 91.7%<br><br>image analysis - 90.8%	Not available	Not available	Not available	Not available

Vandenberghe, 2017<br><br>[48]	Authors proposed DL method (ConvNets) for HER2 cell assessment, evaluated the performance and compared to classical machine learning techniques (Hand-crafted features + Linear Support Vector Machine (LSVM); Hand-crafted features + Random Forests (RF),	Private dataset:<br><br>71 WSI (AstraZeneca BioBank or acquired from a commercial provider (Dako Denmark A/S): (Negative) 43, (Equivocal) 17, (Positive) 11.<br><br>ConvNets<br><br>Deep learning	For 4 classes, WSI level.<br><br>10-fold cross-validation classification performance:<br><br>ConvNets<br>Overall Accuracy 78%	Not available	Not available	In validation:<br>(1+) - 0.80<br>(2+) - 0.58<br>(3+) 0.78	Not available
Pedraza, 2024<br><br>[50]	Color transfer for data augmentation was employed on the initial dataset (DS1) to create a new dataset (DS2) with five classes: background, 0, 1, 2+, and 3+. Additionally, a separate dataset (DS3) was created with seven classes, including 1.5+ and 2.5+. The results from DS3 were then merged back into five classes for comparison. Multiple CNNs were applied for patch-wise grading of HER2.	AIDPATH<br>306 WSIs from 153 BC from 3 centers:<br>172 WSI from NHS (Warwick);<br>104 WSI from SESCAM;<br>30 WSI from SAS<br><br>**(0)** 78<br>**(1)** 74<br>**(2)** 76<br>**(3)** 78<br><br>DL - five different CNNs (The AlexNet, GoogleNet, VGG, ResNet-101, DenseNet-201)	Average accuracy – 97%<br><br>DenseNet-201 on DS2 (dataset 2 – 5 classes: background, 0,1,2,3; balanced, augmented)<br>**(0)** 0.946<br>**(1)** 0.944<br>**(2)** 0.959<br>**(3)** 0.976<br><br>Best accuracy (4 classes of HER2, WSI) - ResNet in DS3 aggregating to 5 classes<br>**(0)** 0.955<br>**(1)** 0.947<br>**(2)** 0.966	Precision<br><br>for DenseNet-201 applied to DS2 dataset with 5 classes.<br><br>**(0)** 0.872<br>**(1)** 0.870<br>**(2)** 0.885<br>**(3)** 0.947<br><br>ResNet in DS3 aggregating to 5 classes<br>**(0)** 0.878<br>**(1)** 0.876<br>**(2)** 0.918	Not available	F1-score<br><br>for DenseNet-201 applied to DS2 dataset with 5 classes.<br><br>**(0)** 0.862<br>**(1)** 0.857<br>**(2)** 0.940<br>**(3)** 0.954<br><br>ResNet in DS3 aggregating to 5 classes<br>**(0)** 0.889<br>**(1)** 0.865<br>**(2)** 0.915	Not available

			**(3)** 0.987<br>Back.- 0.983<br><br>ResNet-101 applied to DS3 dataset with 7 classes (dataset 3 – 7 classes: background, 0,1, 1.5, 2, 2.5, 3)<br>**(0)** 0.968<br>**(1)** 0.954<br>(1.5) 0.975<br>**(2)** 0.974<br>(2.5) 0.986<br>(3) 0.988	**(3)** 0.965		**(3)** 0.968	
Kabakçı 2021<br><br>[51]	Hybrid Cell Detection and Membrane Intensity Histogram Extraction methods were sequentedly used for HER2 scoring, with testing on publick and clinical datasets, and results were compared with Immunomembrane	Mixed (Clinical – the ITU-MED-1, the ITU-MED-2; Warwick)<br>The ITU-MED-1: 13 cases/191 tissue images:<br>(0) 41<br>(1) 42<br>(2) 52<br>(3) 56<br><br>the ITU-MED-2: 10 cases/148 tissue images:<br>(0) 24<br>(1) 18<br>(2) 49<br>(3) 57<br><br>Warwick – 79 WSI	Accuracy (4 classes, patch based): 91.43%<br><br>ITU-MED-1,<br>Best validation accuracy:<br>88.01% (LSTM),<br><br>Best tissue based scoring accuracy: 91.43% (Ensemble Boosted Trees);<br>Compared with 74.07% (ImmunoMembrane);<br><br>ITU-MED-2,<br>Best validation accuracy:<br>88.88% (LSTM),	Tissue based, on ITU-MED:<br>Overall presicion: 91.23%	Tissue based, on ITU-MED:<br>Overall recall: 91.80%	F1-score: 91.81%	Not available

		DL (LSTM);<br>ML (k-Nearest Neighbors (kNN), Decision tree classifiers) for classification.	Best tissue based scoring accuracy: 90.19% (Ensemble Boosted Trees, Ensemble Bagged Trees. Weighted kNN);<br>Compared with 80.39% (ImmunoMembrane).				
Rashid, 2024<br><br>[52]	Combination of transfer learning model (ResNet50) for features extacting, a metaheuristic optimizer (NSGA-II) for selecting most relevant featutes, and a machine learning algorithm (SVM) for classification applied and tested on 2 datasets.	Mixed (Warwick, Clinical):<br>Warwick (HER2SC) 79WSI<br><br>Clinical - 126 individuals (HER2GAN):<br><br>**(0)** 32<br>**(1)** 40<br>**(2)** 30<br>**(3)** 24<br>Transfer Learning	Best accuracy (4 classes, patch level) (Resnet50+NSGA-II+SVM):<br><br>94.4% - on HER2SC<br>90.75% -on HER2GAN	Precision: 0.87	Recall: 0.85	F1-score: 0.86	Not available
Roshan, 2020<br>[26]	Digital image analysis using a free web application	Clinical dataset:<br>60 samples of (2+) HER2/ 307 images	Accuracy 86 %,<br>Specificity 97.8%	Not available	Recall: 46.1%	Not available	Not available
Marcuzzo, 2016<br><br>[53]	Surgical samples and core biopsies were prepared for digital analysis by VISIA Imaging and result were compared with FISH results.	Clinical dataset:<br>176 cases:<br><br>132 (75%) surgical specimens<br>44 (25%) biopsies.<br><br>Negative (1+/0) 23	Sensitivity/ specificity (3 classes, WSI level)<br><br>100% / 82%	Not available	Not available	Not available	Not available

		Equivocal (2+) 85<br>Positive (3+) 44<br>Inadequate 24<br><br>specific software package –<br>VISIA Imaging s.r.l. software (version 2.5.0.1, San Gio-vanni Valdarno, Italy)					
Shovon, 2023<br><br>[54]	Several popular deep learning architectures were employed for feature extraction and classification. Various activation functions were utilized to achieve better results. The classification results of the model trained on H&E and IHC images were compared.	BCI dataset:<br>4870 image pairs with a resolution of 1024*1024 of H&E and IHC, equal number images of HER2 0, 1+, 2+, 3+.<br><br>DL	Best accuracy (4 classes, patch level) DenseNet201-Xception-SIE:<br>97.56% (on IHC data)<br><br>97.12% (on H&E data)	Best Precision DenseNet201-: IHC data<br>97.57%<br><br>H&E data:<br>97.15%	Recall:<br>IHC data<br>98%<br><br>H&E data: 97.68%	Not available	Not available
