# Supplementary material for: The Performance and Clinical Applicability of HER2 Digital Image Analysis in Breast Cancer: A Systematic Review
Source: Cancers (Basel). 2024 Aug 3;16(15):2761. doi: 10.3390/cancers16152761 (PMC11311684; doi:10.3390/cancers16152761)
Supplement: Supplementary file 1 [file cancers-16-02761-s001.zip › Supplemental Table S3-Augmentation.pdf]

Dunenova G et al., "The Performance and Clinical Applicability of HER2 Digital Image Analysis in Breast Cancer: A Systematic Review"

Supplemental Table S3. Data augmentation techniques used

Author	Geometric Augmentation	Intensity and Color Augmentation:	Specialized Transformation	Manual Synthesis of Images	Feature Augmentation in Color Space	Image Processing Augmentation
(Kabir *et al.*, 2024)	Scaling<br>Translation<br>Rotation<br>Vertical Flipping<br>Horizontal Flipping	-	-	-	-	-
(Bórquez *et al.*, 2023)	Random flipping	-	-	-	-	-
(Mukundan, 2019)	-	-	-	-	Transforming to grey-level in CIE-Lab. Augmenting with entropy and energy of the transformed grey-level image.	-
(Tewary and Mukhopadhyay, 2022)	Width Shift<br>Height Shift<br>Shear<br>Horizontal Flip<br>Vertical Flip<br>Rotation	-	-	-	-	-
(Pham *et al.*, 2023)	Rotations<br>Flipping	Brightness and contrast variation<br>Hue and saturation shift	-	-	-	Blurring
(Che *et al.*, 2023)	Rotation<br>Random Flipping	-	-	-	-	-
(Yao *et al.*, 2022)	Random Rotation (-180° to +180°)	-	-	Drawing masks.	-	-

	Random Crop (512, 512)<br>Random Horizontal Flip<br>Random Vertical Flip			Pasting masked parts to create new samples.		
(Yue et al. 2021)	Scale<br>Translation<br>Rotation<br>Flipping	Smoothing	Grammar Transform	-	-	-
(Tewary and Mukhopadhyay, 2021)	Width Shift<br>Height Shift<br>Shear<br>Horizontal Flip<br>Vertical Flip<br>Rotation	-	-	-	-	-
(Pedraza *et al.*, 2024)	Spatial transformations	Colour transfer	-	-	-	-
(Kabakçı *et al.*, 2021)	Rotation<br>Flipping<br>Zooming	Brightness Adjustment	-	-	-	-
(Rashid *et al.*, 2024)	Rotation<br>Flipping<br>Scaling	-	-	-	-	-
(Shovon *et al.*, 2023)	Width Shift<br>Height Shift<br>Vertical Flip	-	-	-	-	-

«-» not available

Abbreviations: CIE-Lab, Commission Internationale de l´Eclairage (International Commission on Illumination's
